# MicroRNAs 146a/b-5 and 425-3p and 24-3p are markers of antidepressant response and regulate MAPK/Wnt-system genes

**DOI:** 10.1038/ncomms15497

**Published:** 2017-05-22

**Authors:** Juan Pablo Lopez, Laura M. Fiori, Cristiana Cruceanu, Rixing Lin, Benoit Labonte, Hannah M. Cates, Elizabeth A. Heller, Vincent Vialou, Stacy M. Ku, Christophe Gerald, Ming-Hu Han, Jane Foster, Benicio N. Frey, Claudio N. Soares, Daniel J. Müller, Faranak Farzan, Francesco Leri, Glenda M. MacQueen, Harriet Feilotter, Kathrin Tyryshkin, Kenneth R. Evans, Peter Giacobbe, Pierre Blier, Raymond W. Lam, Roumen Milev, Sagar V. Parikh, Susan Rotzinger, Steven C. Strother, Cathryn M. Lewis, Katherine J. Aitchison, Gayle M. Wittenberg, Naguib Mechawar, Eric J. Nestler, Rudolf Uher, Sidney H. Kennedy, Gustavo Turecki

**Affiliations:** 1 Department of Psychiatry, McGill Group for Suicide Studies, Douglas Mental Health University Institute, McGill University, Montreal, Quebec, Canada H4H 1R3; 2 Fishberg Department of Neuroscience and Friedman Brain Institute, Icahn School of Medicine at Mount Sinai, New York, New York 10029, USA; 3 Department of Psychiatry, University Health Network, University of Toronto, Toronto, Ontario, Canada M5T 2S8; 4 McMaster University and St Joseph's Healthcare Hamilton, Hamilton, Ontario, Canada L8S 4L8; 5 St Michael's Hospital, Toronto, Ontario, Canada M5B 1M4; 6 Department of Psychiatry, Queen's University, Kingston, Ontario, Canada K7L 3N6; 7 Centre for Addiction and Mental Health, Toronto, Ontario, Canada M6J 1A8; 8 School of Mechatronic Systems Engineering, Surrey, British Columbia, Canada V3T 0A3; 9 University of Guelph, Guelph, Ontario, Canada N1G 2W1; 10 University of Calgary Hotchkiss Brain Institute, Calgary, Alberta, Canada T2N 1N4; 11 Department of Pathology and Molecular Medicine, Queen's University, Kingston, Ontario, Canada K7L 3N6; 12 Indoc Research, Toronto, Ontario, Canada M5A 1N1; 13 University of Ottawa Institute of Mental Health Research, Ottawa, Ontario, Canada K1Z 7K4; 14 University of British Columbia and Vancouver Coastal Health Authority, Vancouver, British Columbia, Canada V6T 2A1; 15 Queen's University, Providence Care, Mental Health Services, Kingston, Ontario, Canada K7L 4X3; 16 University of Michigan, Ann Arbor, Michigan 48109, USA; 17 Rotman Research Institute at Baycrest Centre, Toronto, Ontario, Canada M6A 2E1; 18 MRC Social, Genetic and Developmental Psychiatry Centre, Institute of Psychiatry, Psychology & Neuroscience, King's College London, London SE5 8AF, UK; 19 Departments of Psychiatry and Medical Genetics, University of Alberta, Edmonton, Alberta, Canada T6G 2B7; 20 Department of Psychiatry, Dalhousie University, Halifax, Nova Scotia, Canada B3H 2E2; 21 Janssen Research & Development, LLC, San Diego, California 92121, USA

## Abstract

Antidepressants (ADs) are the most common treatment for major depressive disorder (MDD). However, only ∼30% of patients experience adequate response after a single AD trial, and this variability remains poorly understood. Here, we investigated microRNAs (miRNAs) as biomarkers of AD response using small RNA-sequencing in paired samples from MDD patients enrolled in a large, randomized placebo-controlled trial of duloxetine collected before and 8 weeks after treatment. Our results revealed differential expression of miR-146a-5p, miR-146b-5p, miR-425-3p and miR-24-3p according to treatment response. These results were replicated in two independent clinical trials of MDD, a well-characterized animal model of depression, and post-mortem human brains. Furthermore, using a combination of bioinformatics, mRNA studies and functional *in vitro* experiments, we showed significant dysregulation of genes involved in *MAPK/Wnt* signalling pathways. Together, our results indicate that these miRNAs are consistent markers of treatment response and regulators of the *MAPK/Wnt* systems.

## Abstract (web summary)

Antidepressant drugs are the most common treatment for depressive episodes but only a fraction of patients experience adequate response. Here the authors find dysregulation of miRNAs in peripheral blood samples from depressed patients after antidepressant treatment, and show that the miRNAs are regulators of psychiatrically relevant signalling pathways.

Major depressive disorder (MDD) is a prevalent illness that associates with significantly increased mortality, disability and secondary morbidity, and is one of the world's leading causes of disease burden^1^. Treatment of MDD includes a variety of biopsychosocial approaches, but in medical practice, antidepressant (AD) drugs are the most common treatment for depressive episodes and, not surprisingly, they are among the most prescribed medications in the world^2^. While they are clearly effective, particularly for moderate-to-severe depressive episodes, there is substantial variability in how individuals respond to AD treatment. In spite of the many ADs available, almost 70% of patients do not respond to a single trial and 30–40% of patients do not present with a full response following several trials^3^. The failure to respond has important individual, economic and social consequences for both patients and their families. Thus, there is a great need to better understand factors associated with response to AD treatment.

Genes can be regulated through the activity of several noncoding RNA transcripts that act as fine-tuners and on–off switches of gene expression patterns^4^. Among the noncoding RNAs, microRNAs (miRNAs) have been the most extensively studied^5^. Since their discovery in the early 1990s, miRNAs have revolutionized our understanding of gene regulation and show great potential for assisting in the elucidation of mechanisms underlying disease pathology and treatment response^6^. MiRNAs are small noncoding, single-stranded RNA transcripts that regulate the expression of mRNAs through RNA degradation or translational repression. There is increasing evidence suggesting a key role for miRNAs in the regulation of essential processes of brain functioning, as well as in the development of psychiatric disorders and their treatments^7^. For instance, Baudry *et al*.^8^,^9^ found that miR-16 mediates AD action of fluoxetine in the raphe and locus coeruleus of mice, and follow-up work suggested that this same miRNA displays antidepressant-like effects and mediates adult neurogenesis in the hippocampus. Another recent and promising finding uncovered a key role for miR-135a in the molecular mechanisms underlying the therapeutic actions of ADs in both depressed humans and animal models of depression^10^. Dias *et al*.^11^ showed that the miRNA processing protein, Dicer, acts in brain reward regions to promote antidepressant-like responses in animal models. Finally, we have recently reported that miR-1202, a primate-specific and brain-enriched miRNA, is downregulated in the prefrontal cortex of depressed individuals and regulates the expression of the *metabotropic glutamate receptor* (*GRM4*)^12^. These encouraging new findings underline the potential of studying miRNAs to better understand molecular processes associated with MDD. Here, we investigate miRNAs in relation to AD response. Using complementary strategies, we demonstrate that levels of miR-146a-5p, miR-146b-5p, miR-24-3p and miR-425-3p are modified as a function of AD response and regulate genes involved in mitogen-activated protein kinase (MAPK) and Wnt signalling pathways.

## Results

### MicroRNA dysregulation after antidepressant treatment

**Figure 1:**
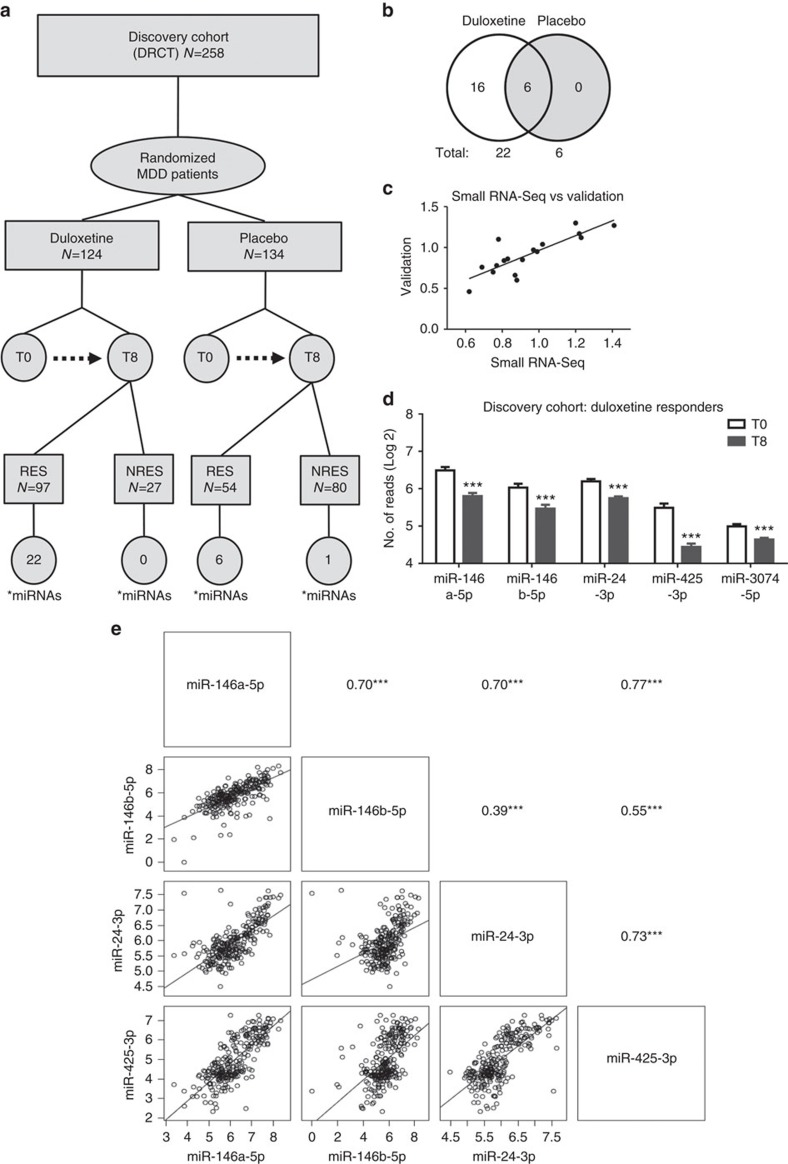
MicroRNA changes after 8 weeks of AD treatment. (**a**) Experimental design. Discovery cohort (DRCT), baseline (T0), 8 weeks of treatment (T8), responders (RES) and nonresponders (NRES), *Number of significant miRNAs. (**b**) Venn diagram showing specific and commonly dysregulated miRNAs in duloxetine and placebo responders. (**c**) Correlation between miRNAs measured by small RNA-sequencing and a high-sensitivity multiplex cellular miRNA assay on a standard flow cytometer (Firefly BioWorks). (**d**) Small RNA Sequencing expression (number of reads per million, Log2) of miR-146a-5p, miR-146b-5p, miR-24-3p, miR-425-3p and miR-3074-5p after 8 weeks in MDD patients who responded to duloxetine treatment. ***FDR corrected *P*<0.001. (**e**) Scatterplots of Pearson's correlations between differentially expressed miRNAs in blood samples from the discovery cohort. ***P<0.001.

We performed small RNA-sequencing in blood samples from a randomized placebo-controlled trial of duloxetine, a serotonin–norepinephrine reuptake inhibitor. Blood samples were collected before and 8 weeks after treatment (discovery cohort (DRCT); *n*=516 samples from 258 patients) (Fig. 1a). We compared the expression of miRNAs before and after treatment (that is, 8-week response to baseline). Statistical analysis, using Benjamini–Hochberg correction for multiple testing (5% false discovery rate (FDR)) and corrected *P* values of <0.05, revealed differential expression of 22 miRNAs after 8 weeks of duloxetine treatment (Table 1) and 6 miRNAs after 8 weeks of placebo treatment (Table 2). Except for one miRNA, these findings were specific to patients who responded to treatment with no differences found in the nonresponder groups (Fig. 1a and Supplementary Tables 1 and 2). We defined response as >50% decrease in Montgomery–Åsberg Depression Rating Scale (MADRS) scores from baseline to trial end point^13^,^14^,^15^. Remarkably, the six miRNAs that were significantly changed according to placebo response were among the 22 whose expression changed according to duloxetine response, and were altered in the same direction (Tables 1 and 2; Fig. 1b). Thus, 16 miRNAs changed specifically according to duloxetine treatment. One miRNA (miR-503-5p) was significantly changed in responders and nonresponders in both placebo- and duloxetine-treated patients, suggesting that the alterations in the expression of this miRNA may associate with metabolic processes that are independent of clinical outcome.

We validated our significant findings using a high-sensitivity multiplex cellular miRNA assay on a standard flow cytometer (Firefly BioWorks). Using a custom panel, we measured miRNA expression before and after treatment in the same 516 samples used for small RNA-sequencing. We validated 87% of the miRNAs tested (Supplementary Table 3) and found a significant correlation of fold changes across the two technologies (*r*=0.83, *P*<0.05; Fig. 1c). Since we were interested in the underlying molecular mechanisms involved in response to treatment, and based on recent evidence suggesting that some of the mechanisms involved in placebo response are also engaged during AD treatment and could reinforce response to common AD^16^,^17^, we next focused on the miRNAs that were differentially expressed according to response in both the duloxetine and placebo groups. These were miR-146a-5p, miR-146b-5p, miR-24-3p, miR-425-3p and miR-3074-5p that were all downregulated after treatment, and their individual expressions were strongly correlated (Fig. 1d,e and Supplementary Table 4) suggesting a shared mechanism of action.

### Independent replication with cohort 1

**Figure 2:**
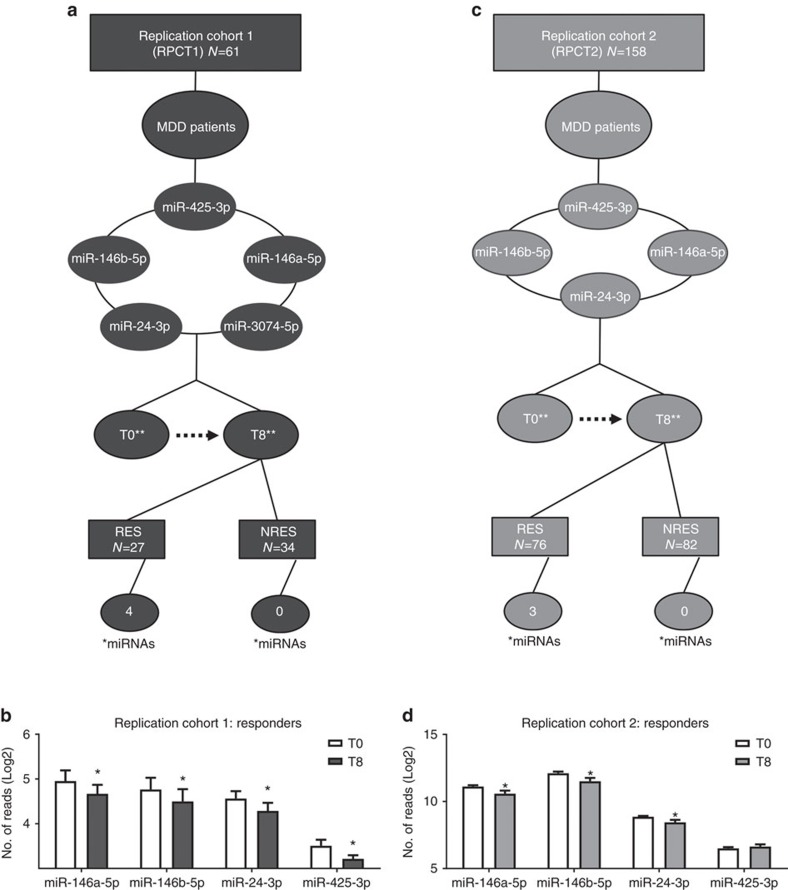
Independent replication cohorts. (**a**,**c**) Experimental design. Replication cohorts (RPCT1 and RPCT2), baseline (T0), 8 weeks of treatment (T8), responders (RES) and nonresponders (NRES). *Number of significant miRNAs. **Patients were treated with escitalopram or nortriptyline. (**b**,**d**) Log2 expression of miRNAs after 8 weeks of antidepressant treatment. *P<0.05.

To test the external validity of our findings, we measured the expression of these five miRNAs before and after 8 weeks of AD treatment in a fully independent sample composed of 61 depressed patients (122 samples) who were recruited as part of the Genome-based Therapeutic Drugs for Depression (GENDEP) study^18^ (replication cohort 1). Patients were treated with escitalopram, a selective serotonin reuptake inhibitor, or nortriptyline, a tricyclic antidepressant (Fig. 2a). In line with our findings in the DRCT, we again found a significant downregulation in the expression of miR-146a-5p, miR-146b-5p, miR-24-3p and miR-425-3p in the blood of depressed patients after treatment. Importantly, these changes were consistent with those observed in the discovery cohort (Fig. 2b and Supplementary Table 5). Although we also saw a decrease in the expression of miR-3074-5p, this effect was not statistically significant, and thus we did not include this miRNA in further analyses. Consistent with our initial findings, the downregulation of the validated miRNAs was only observed among responders to treatment, as we found no changes in the expression of these miRNAs in the nonresponder group. These findings support a relationship between treatment response and miR-146a-5p, miR-146b-5p, miR-24-3p and miR-425-3p.

### Independent replication with cohort 2

We measured the expression of miR-146a-5p, miR-146b-5p, miR-24-3p and miR-425-3p before and after 8 weeks of AD treatment in a second independent sample composed of 316 additional blood samples from 158 patients treated with the antidepressant escitalopram (selective serotonin reuptake inhibitor) for 8 weeks. All patients from cohort 2 (RPCT2) were recruited as part of the Canadian Biomarker Integration Network in Depression (CAN-BIND) study^19^. (Fig. 2c). In line with all our previous findings, we found consistent results, both in significance and direction, for three out of the four miRNAs tested (Fig. 2d and Supplementary Table 6). There was a downregulation of miR-146a-5p, miR-146b-5p and miR-24-3p after antidepressant treatment. More importantly, these effects were only observed among responders to escitalopram treatment, as we found no changes in the expression of these miRNAs in the nonresponder group. We found no significant differences in the levels of miR-425-3p in our second replication cohort (Fig. 2d and Supplementary Table 6). These findings support a relationship between these miRNAs and treatment response in humans.

### Ruling out effects of differential blood cellular composition

To control for possible biases arising from differential blood sample cellular composition between groups, we used haematology data collected in the discovery sample during the randomized placebo-controlled trial. We found no significant differences between groups or categories (before and after treatment, between responders and nonresponders, and between duloxetine- and placebo-treated individuals) in the levels or proportions of any of the cell types investigated in the blood samples from either duloxetine-treated patients or placebo controls (Supplementary Tables 7 and 8). More importantly, we measured the levels of the validated and replicated miRNAs in cell-free plasma samples obtained from these subjects, and observed results that were similar to those observed with the whole blood samples, both in significance and direction (Supplementary Fig. 1).

### Dysregulation of miRNAs after antidepressant treatment in mice

**Figure 3:**
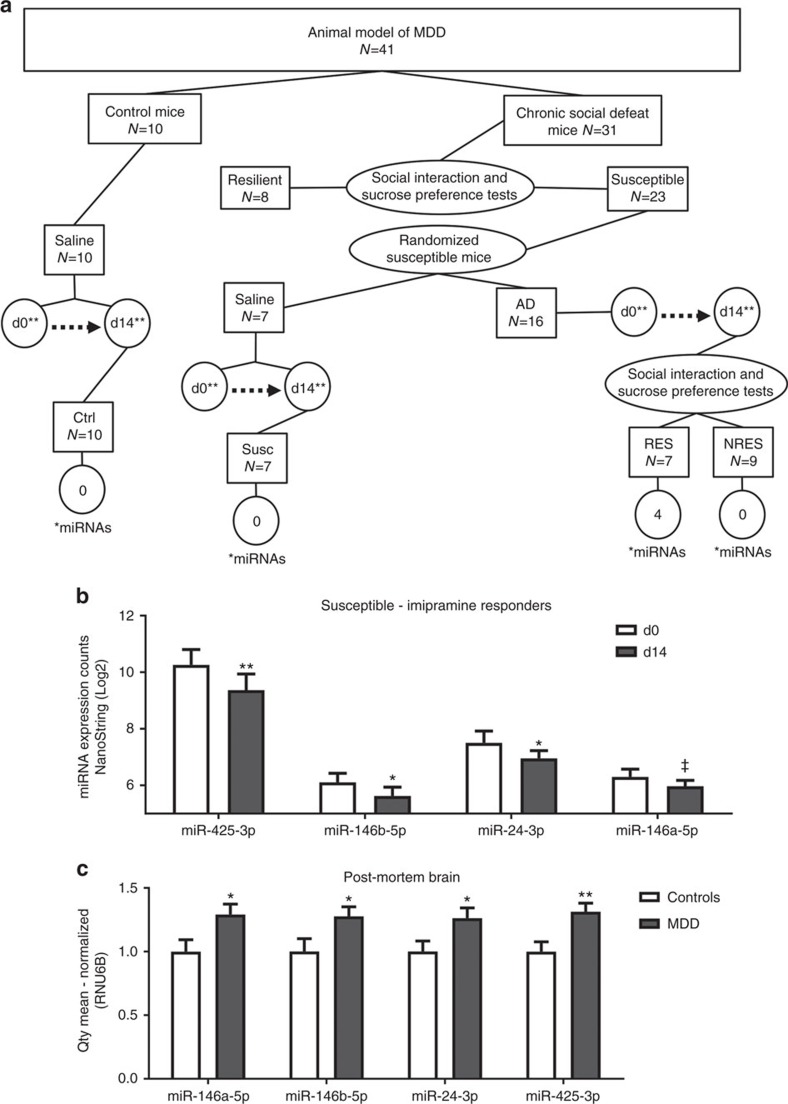
Expression of miR-146a-5p, miR-146b-5p, miR-24-3p and miR-425-3p in an animal model of MDD and post-mortem human brain samples. (**a**) Experimental design. Mouse model of MDD. Baseline (d0), 14 days of treatment (d14), responders (RES), nonresponders (NRES), susceptible (Susc) and Control (Ctrl). *Number of significant miRNAs. **Mice were treated with imipramine or saline solution. Resilient mice were not studied. (**b**) Differential expression of miR-146a-5p, miR-146b-5p, miR-24-3p and miR-425-3p after 14 days of imipramine treatment in mice by NanoString nCounter Digital Analyzer. *P<0.05; **P<0.01; ^‡^*P*<0.1. (**c**) Quantitative real-time PCR (qRT-PCR) expression data of miR-146a-5p, miR-146b-5p, miR-24-3p and miR-425-3p in vPFC of post-mortem brains of subjects with MDD and controls. *P<0.05; **P<0.01.

We next investigated the relevance of our results in a well-established animal model of depression^20^. We tested mice exposed to the chronic social defeat stress (CSDS) paradigm followed by the social interaction and sucrose preference tests to distinguish between susceptible animals, which show deficits in these behaviours, and resilient animals, which do not^20^. Susceptible mice were then treated with the antidepressant imipramine (tricyclic antidepressant) or saline control for 14 days followed by a repeat of the social interaction and sucrose preference tests. Mice that showed improvement in depression-like symptoms in these behavioural assays were classified as responders, while those that showed no improvements were deemed as nonresponders (Fig. 3a). We measured the expression of miR-146a-5p, miR-146b-5p, miR-24-3p and miR-425-3p in peripheral blood samples of mice before and after treatment with imipramine or saline control, using a NanoString, a probe-based miRNA expression assay. We found a significant downregulation of miR-146b-5p, miR-24-3p and miR-425. In addition, we found a decrease in the expression of miR-146a-5p, but these findings did not reach statistical significance (*P*=0.09; Fig. 3b). Consistent with the data in humans, these changes were selective for the responder group, as no differences were detected in the nonresponders or saline control groups (Table 3). These results reinforce the relationship between these miRNAs and treatment response.

### Dysregulation of miRNAs in the vPFC of depressed individuals

To explore the relationship between the peripheral changes of miR-146a-5p, miR-146b-5p, miR-24-3p and miR-425-3p, and their expression in the brain, we used quantitative real-time PCR to assess the levels of these miRNAs in the ventrolateral prefrontal cortex (vPFC) of depressed humans who died by suicide (*n*=32) and psychiatrically healthy controls (*n*=20). We found a significant difference in the expression of each of these miRNAs compared with controls (Fig. 3c and Table 4). Whereas the peripheral samples from AD treatment responders showed decreased expression of the candidate miRNAs, these miRNAs were all upregulated in depressed brain tissue relative to healthy controls (Fig. 3c and Table 4). Additionally, their expression was strongly correlated in brain tissue, again suggesting a common mechanism of action (Supplementary Fig. 2 and Supplementary Table 9).

### miRNA target prediction and pathway analysis

**Figure 4:**
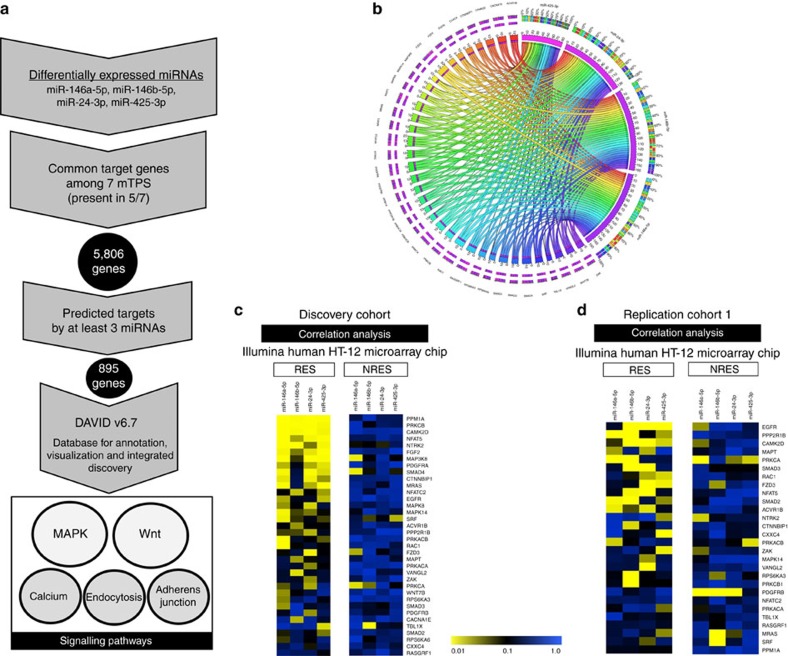
Target prediction analysis and miRNA regulation of MAPK and Wnt signalling after antidepressant treatment. (**a**) Target prediction analysis using seven miRNA target prediction sites (mTPS): miRWalk 2.0, miRDB, miRanda, RNA22 v2, RNAHybrid, TargetScan and DIANA Tools. Biological pathway analysis using the Database for Annotation, Visualization and Integrated Discovery (DAVID) v6.7. (**b**) Circo graph shows the relationship between miR-146a-5p, miR-146b-5p, miR-24-3p and miR-425-3p with 36 predicted MAPK and Wnt genes identified through our *in silico* analysis. Each ribbon connects a miRNA with a predicted target gene. (**c**,**d**) Correlation analysis between miR-146a-5p, miR-146b-5p, miR-24-3p, miR-425-3p and MAPK/Wnt genes in the discovery and replication cohorts. Heatmap represents *P* values of Pearson's correlations.

Gene targets of miR-146a-5p, miR-146b-5p, miR-24-3p and miR-425-3p were identified using seven miRNA target prediction databases. We opted to consider only targets that were commonly predicted by at least 5 out of the 7 prediction databases, and obtained a list of 5,806 putative target genes (Fig. 4a). As the changes of these 4 miRNAs were positively correlated, we hypothesized that they had common pathways of regulation, and thus narrowed our list of targets by considering only genes that were commonly predicted targets for at least 3 of the miRNAs, producing a list of 895 genes. We then used the Database for Annotation, Visualization and Integrated Discovery (DAVID) v6.7 (ref. 21) to identify biological pathways that were potentially regulated by these miRNAs. Functional Annotation Clustering analysis revealed an enrichment of five signalling pathways. The *MAPK*, *Wnt*, calcium, endocytosis and adherens junction signalling pathways were significantly enriched after Benjamini–Hochberg correction for multiple testing (*P*<0.05; Fig. 4a,b). To validate our miRNA target prediction and pathway analysis, we performed a second, independent *in silico* analysis using DIANA: miRPath^22^. Consistent with our first *in silico* analysis, we found a significant enrichment of the *MAPK*, *Wnt*, calcium, endocytosis and adherens junction signalling pathways after FDR correction for multiple testing (*P*<0.05). Interestingly, the *MAPK* and *Wnt* signalling pathways are modulated by stress and have been previously implicated in MDD and treatment response in humans and animal models^11^,^23^,^24^,^25^.

### MiRNA regulation of MAPK/Wnt signalling after treatment

To explore the relationship between the downregulated miRNAs and predicted signalling pathways, we cross-referenced the expression of miR-146a-5p, miR-146b-5p, miR-24-3p and miR-425-3p with the genes identified through our computational analysis. Given the previous evidence suggesting changes in the *MAPK* and *Wnt* signalling pathways in depression, we focused only on these two pathways. First, we used mRNA microarray expression data obtained from blood of the DRTC samples (*n*=460 samples from 230 patients). Interestingly, we found that most of the predicted target genes involved in *MAPK* and *Wnt* signalling pathways were significantly correlated with the expression of one or more of the miRNAs tested (89%) (Supplementary Table 10). However, this effect was significantly stronger in patients who responded to treatment (Fig. 4c and Supplementary Table 11). In addition, 69% of these genes were significantly dysregulated following duloxetine treatment (Supplementary Fig. 3). We then performed a similar analysis using complementary miRNA and gene expression data obtained from our replication cohort (*n*=102 samples from 51 patients). We consistently found a significant correlation between predicted genes involved in *MAPK* and *Wnt* signalling pathways and the expression of the downregulated miRNAs in depressed patients who responded to treatment (71%) (Supplementary Table 12). Once again, the effect was stronger in the responder group as compared with the nonresponder group (Fig. 4d). To verify that the correlation between these miRNAs and the *MAPK/Wnt* genes identified through our *in silico* analysis was not a random finding, we performed a permutation analysis to test correlations between miR-146a-5p, miR-146b-5p, miR-24-3p, miR-425-3p and 50 random genes. We found that the average percentage of significant correlations with 50 random genes after 100 permutations was 12% for all miRNAs tested, suggesting that the results found in our discovery (89%) and replication (71%) cohorts were significantly above chance levels. Together, these results support a relationship between these miRNAs and the *MAPK/Wnt* signalling pathways after antidepressant treatment.

### MicroRNA dysregulation of *MAPK/Wnt* signalling *in vitro*

**Figure 5:**
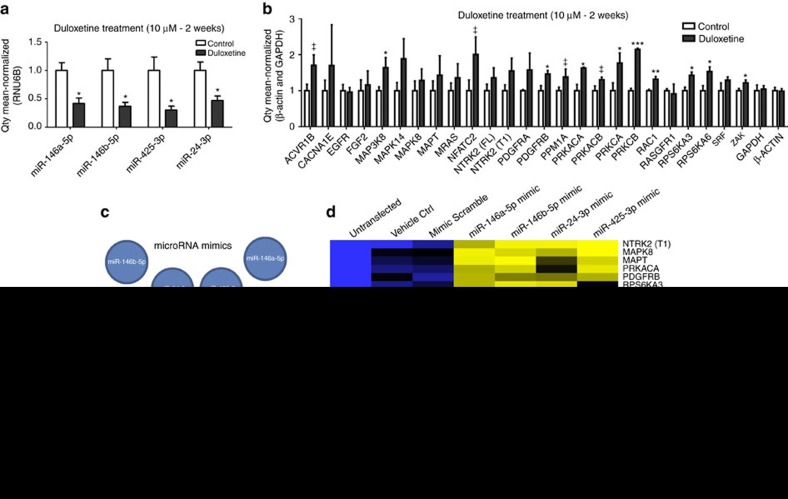
MicroRNA dysregulation of MAPK and Wnt signalling pathways. (**a**) Expression of miR-146a-5p, miR-146b-5p, miR-24-3p and miR-425-3p in human NPCs treated with duloxetine or a no-drug control for 2 weeks (chronic treatment). *P<0.05. (**b**) Expression of MAPK/Wnt target genes in human NPCs treated with duloxetine or a no-drug control for 2 weeks (chronic treatment). *P<0.05; **P<0.01; ***P<0.001; ^‡^*P*<0.1. (**c**) Overexpression of miRNAs using miRNA mimics on human embryonic kidney cells (HEK293). (**d**) Heatmap showing *P* values of MAPK/Wnt target genes after overexpression of miRNAs in HEK293 cells.

To confirm that antidepressants have an effect on the expression of miR-146a-5p, miR-146b-5p, miR-24-3p and miR-425-3p, we treated human neural progenitor cells (NPCs) with duloxetine, the same antidepressant that was used in the DRTC, as well as a no-drug control. NPCs display a serotonergic phenotype and express endogenous levels of the *serotonin transporter* (*SERT*) and several other serotonin receptors^12^. Following chronic duloxetine treatment for 2 weeks, we found a significant downregulation of miR-146a-5p, miR-146b-5p, miR-24-3p and miR-425-3p (Fig. 5a), with no differences in the expression of the endogenous control *RNU6B* after treatment or changes in these miRNAs after 2 weeks in untreated cells. In addition, we tested the expression of the predicted target genes involved in *MAPK* and *Wnt* signalling pathways that were correlated in our discovery and replication cohorts. We found a dysregulation in the expression of most (92%) *MAPK/Wnt* genes tested after chronic duloxetine treatment, but found no significant differences in the expression of the endogenous controls *β-Actin* and *GAPDH*. Most importantly, and consistent with our miRNA findings, we found an upregulation of these genes after treatment as compared with controls (Fig. 5b). These results support an interaction between these four miRNAs, the *MAPK/Wnt* signalling pathways and antidepressant treatment.

Finally, to experimentally confirm the interaction between miR-146a-5p, miR-146b-5p, miR-24-3p, miR-425-3p and genes of the *MAPK/Wnt* signalling pathways, we performed functional experiments using miRNA mimics on human embryonic kidney cells (HEK293). The HEK293 cells showed relatively high levels of all genes tested, but low levels of the miRNAs tested and thus provided a good model for our functional assays. We examined whether overexpression of any of these miRNAs affects the expression of *MAPK/Wnt* genes *in vitro*. Cells were transfected for 24 h with a miRNA mimic for each of the individual miRNAs (miR-146a-5p, miR-146b-5p, miR-24-3p and miR-425-3p), a miRNA-mimic scramble control or a mock vehicle, and results were compared with untreated cells. A one-way analysis of variance showed that all of the *MAPK/Wnt* genes tested were downregulated by at least one of the miRNA mimics transfected (Fig. 5c,d). Seven of the tested genes were downregulated by each of the four miRNA mimics. Thirteen genes were downregulated by three of the four miRNA mimics. Two genes were downregulated by two of the four miRNA mimics and one gene was only downregulated by one of the four miRNA mimics (Fig. 5c,d and Supplementary Table 13). We found no effects on any of these genes after treatment with the scramble or vehicle controls. In addition, we did not find any changes in the expression of *GAPDH* or *β-Actin* after transfection of the mimics, scramble or vehicle controls. These results confirm an interaction between miR-146a-5p, miR-146b-5p, miR-24-3p, miR-425-3p and the *MAPK/Wnt* signalling pathways.

## Discussion

These miRNAs are transcribed from different genomic loci: chr5 (miR-146a-5p), chr10 (miR-146b-5p), chr9/chr19 (miR-24-3p) and chr3 (miR-425-3p). They are ubiquitously expressed, highly correlated in blood and brain tissue, and also expressed in other human biological fluids (Supplementary Figs 4 and 5). Based on our results, we propose a potential mechanism of action for these miRNAs through regulation of the *MAPK* and *Wnt* signalling pathways. Interestingly, inhibition of these pathways has been shown to lead to a depressive phenotype, while activation has been strongly associated with a decrease in depressive symptoms and improved antidepressant response in both central and peripheral systems^26^,^27^,^28^,^29^,^30^. Here, we showed that miR-146a-5p, miR-146b-5p, miR-24-3p and miR-425-3p regulate the expression of >30 genes involved in *MAPK* and *Wnt* signalling. Remarkably, most of these genes have been previously associated with MDD or antidepressant activity (Supplementary Table 14). In addition, we found a dysregulation of key genes involved in neurotrophic factor signalling that is directly coupled to *MAPK* activity. *The tropomyosin receptor kinase B* (*NTRK2*) is the preferred receptor of the *brain-derived neurotrophic factor* (*BDNF*) and a key player in the neurotropic hypothesis of depression^31^,^32^, while the *fibroblast growth factor* (*FGF2*) is a vital growth factor recently shown to promote neuronal plasticity, proliferation, survival, differentiation and synaptic function^33^. Both of these genes have been shown to regulate neurogenic mechanisms and antidepressant response^23^,^26^. Other genes involved in calcium signalling, endocytosis and adherens junctions are also potentially regulated by these miRNAs.

In summary, we report a consistent dysregulation of miRNAs in peripheral blood samples from depressed patients after antidepressant treatment. Our findings indicate that miR-146a-5p, miR-146b-5p, miR-24-3p and miR-425-3p are consistent blood markers of antidepressant response and regulators of psychiatrically relevant signalling pathways. More importantly, we showed consistent changes in the expression of these miRNAs in patients who respond to treatment with different classes of ADs, as well as in stressed mice that respond to AD, suggesting their involvement in a common mechanism of action. These results have important implications for the elucidation of biological mechanisms involved in clinically relevant antidepressant effects and suggest that these miRNAs could potentially be used as correlates or state biomarkers of treatment response. Ultimately, our results underline the potential of studying miRNAs to better understand molecular processes associated with MDD and provide valuable information for the development of diagnostic tools, precautionary approaches and successful pharmacological interventions for MDD.

## Methods

### Human samples

A total of 1,006 human samples were included in the present study, and include (1) discovery cohort (DRCT; *n*=516 samples from 258 patients) obtained in collaboration with the CAN-BIND and Lundbeck A/S sponsored clinical trials; (2) replication cohort 1 (RPCT1, *n*=122 samples from 61 patients) obtained in collaboration with the GENDEP consortium; (3) replication cohort 2 (RPCT2, *n*=316 samples from 158 patients) obtained in collaboration with CAN-BIND; and (4) post-mortem, prefrontal cortex brain tissue (*n*=52) that was obtained in collaboration with the Quebec Coroner's Office and the Douglas-Bell Canada Brain Bank (Douglas Mental Health University Institute, Montreal, Canada). Ethics approval for this study was obtained from the institutional review board of the Douglas Mental Health University Institute, and written informed consent was obtained from patients or family members, as appropriate.

Our DRCT consisted of 258 well-characterized individuals (females *n*=178; males=80) diagnosed with MDD in a current major depressive episode (MDE) who were enrolled in a clinical trial and received treatment with either duloxetine or placebo. Duloxetine is a serotonin–norepinephrine reuptake inhibitor. For each patient, peripheral blood samples were collected at baseline and after treatment. Participants, aged 19–74 years, were recruited based on a primary diagnosis of MDD and MDE lasting at least 3 months, with a severity score on the MADRS at baseline of ≥22. Participants resistant to at least two previous AD treatments or who had received electroconvulsive therapy in the 6 weeks before study beginning were excluded. Other exclusion criteria were: MDE in bipolar disorder, presence of psychotic features and recent substance use disorder. Eligible participants were equally randomized to double-blind treatment with either duloxetine (60 mg) or placebo. All participants were assessed prospectively for 8 weeks, including measures of depressive severity and potential related adverse events. To quantify treatment response, we calculated percentage change of MADRS scores (from baseline to after treatment). We used percentage change to correct for the potential effects of differential baseline scores. Additionally, we classified participants as responder/nonresponder based on >50% decrease in MADRS scores from baseline. This project was approved by ethics boards of participating centres, and all participants provided written informed consent. Clinical trials are registered at www.ClinicalTrials.gov (11984A NCT00635219; 11918A NCT00599911; 13267A NCT01140906)

Our RPCT1 consisted of 61 well-characterized individuals (females *n*=40; males=21) diagnosed with MDD and enrolled in the GENDEP project. Adult patients with major depression of at least moderate severity according to ICD-10 (International Statistical Classification of Diseases and Related Health Problems, 10th revision) or DSM-IV (Diagnostic and Statistical Manual of Mental Disorders, 4th Edition) criteria were recruited at five European centres. Patients were aged 21–70 years and of Caucasian European parentage. Exclusion criteria were personal and family history of schizophrenia or bipolar disorder, or current substance dependence. Eligible participants were allocated to treatment with either escitalopram or nortriptyline that represent different antidepressant classes and mechanisms of action. Patients with contraindications for one drug were offered the other. Severity of depression was assessed at baseline and after treatment by three established rating scales. The GENDEP project was approved by ethics boards of participating centres, and all participants provided written informed consent. The GENDEP trial is registered at EudraCT (no. 2004-001723-38) and ISRCTN (no. 03693000).

RPCT2 consisted of 158 well-characterized individuals (females *n*=98; males=60) diagnosed with MDD and enrolled in the CAN-BIND project^19^. Adult patients with major depression of at least moderate severity according to DSM-IV-Text Revision (TR) criteria were recruited at six Canadian clinical centres. Patients were aged 18–60 years. Exclusion criteria were personal and family history of schizophrenia or bipolar disorder, or current substance dependence. Eligible participants were allocated to treatment with escitalopram (10–20 mg per day) for 8 weeks. Severity of depression was assessed at baseline and after treatment by the MADRS. The CAN-BIND project was approved by ethics boards of participating centres, and all participants provided written informed consent. The CAN-BIND trial is registered at ClinicalTrials.gov identifier NCT01655706. Registered 27 July 2012.

Human post-mortem prefrontal cortex brain tissue was obtained in collaboration with the Quebec Coroner's Office and the Douglas-Bell Canada Brain Bank (Douglas Mental Health University Institute, Montreal, Quebec, Canada). A total of 52 brain samples were included in the present study. Thirty two depressed suicide completers and 20 non-suicide controls were randomly selected for miRNA analysis. All individuals were of French–Canadian origin, a homogeneous population with a well-documented founder effect, and were matched for refrigeration delay, age and pH. Refrigeration delay refers to the difference between the estimated time of death (determined by the pathologist through external body examination details) and the time at which the brain was refrigerated. Psychological autopsies were performed post mortem on both cases and controls by a panel of psychiatrists, and diagnoses were assigned based on DSM-IV criteria. The control group had no history of suicidal behaviour or major mood or psychotic disorders.

Peripheral blood samples were collected in PAXgene blood RNA tubes (PreAnalytix). PAXgene tubes were frozen using a sequential freezing process: tubes were stored at room temperature for 3 h, transferred to 4 °C overnight, followed by 6–8 h at −20 °C and then final storage at −80 °C. Total RNA (including the miRNA fraction) was isolated from whole blood using the PAXgene Blood miRNA Kit (Qiagen, Canada) according to the manufacturer's instructions. Brain tissues were processed and dissected at 4 °C, then snap-frozen in liquid nitrogen before storage at −80 °C. Total RNA was isolated from 40 mg of frozen PFC tissue using the miRNeasy Mini Kit protocol in combination with the RNeasy MinElute Cleanup Kit (Qiagen, Canada), with no modifications. All samples were treated with DNase digestion during RNA purification using the RNase-Free DNase kit (Qiagen). RNA and miRNA yield and quality were determined using the Nanodrop 1000 (Thermo Scientific, USA) and Agilent 2100 Bioanalyzer (Agilent Technologies, USA). The average RNA Integrity Number (RIN) value for all our samples by group were as follows: discovery cohort RIN: 8.6, replication cohort 1 RIN: 8.3, replication cohort 2 RIN: 8.2, and post-mortem brain samples RIN: 6.7.

All libraries were prepared using the Illumina TruSeq Small RNA protocol following the manufacturer's instructions with 12 cycles of PCR amplification after ligation of the 3′ and 5′ adapters, as described elsewhere^5^. Libraries were purified using biotinylated magnetic AMPure beads that allow for selection of specified complementary DNA (cDNA) products bound to streptavidin. A total of 50 μl of amplified cDNA were mixed and purified twice with AMPure XP beads in a 1.8:1 ratio (beads/sample). This ratio allows for optimal selection of all products higher than 100 nucleotides. Library qualities were assessed using an Agilent 2100 Bioanalyzer High Sensitivity DNA chip, as well as quantitative real-time PCR with the KAPA library quantification kit (Kapa Biosystems, USA).

Samples were sequenced at the McGill University and Genome Quebec Innovation Centre (Montreal, Canada) using the HiSeq2500 Illumina sequencer with 50-nucleotide single-end reads. We obtained an average number of 12.1 million raw reads, with an average quality value of 37 in all libraries produced. All sequencing data were processed using CASAVA 1.8+(ref. 34) and extracted from FASTQ files. The Fastx_toolkit was used to trim the Illumina adapter sequences. Additional filtering based on defined cutoffs was applied to obtain high-quality data. These filters included: (1) Phred quality (Q) mean scores higher than 30, (2) reads between 15 and 40 nucleotides in length, (3) adapter detection based on perfect 10-nucleotide match and (4) removal of reads without detected adapter. Additionally, we used Bowtie^35^ to align reads to the human genome (GRCh37)^36^ and ncPRO-seq^37^ in combination with miRBase (V20) to match them to known miRNA sequences^38^,^39^. We used a detection threshold of 10 counts per miRNA (present at least in 80% of libraries tested). A total of 281 miRNAs survived our criteria and were included in the analysis. All small RNA-sequencing data were normalized with the Bioconductor – DESeq2 package using the variance-stabilizing transformation method^40^. To identify significant microRNAs based on differential expression changes across time, we used significance analysis of microarrays method, adapted for small RNA-sequencing data (SAMSeq)^41^. SAMSeq was implemented using a two-class paired model that accounts for changes not only between groups, but also within subjects as there is a one-to-one pairing between subjects across time. We used the Benjamini–Hochberg FDR correction for multiple testing set at 5% that corrects for the proportion of miRNAs likely to have been identified by chance as being significant (adjusted *P* values<0.05). We also tested for potential confounding effects of age, gender, race and RIN but found no significant differences across groups or time.

Small RNA-sequencing results were validated with a custom miRNA panel using circulatory miRNA assays by Firefly BioWorks. Briefly, RNA samples were hybridized to miRNA-specific probes coated on FirePlex hydrogel beads. A universal biotinylated adapter was then ligated onto the captured miRNAs and labelled with a fluorescent reporter. The level of fluorescence for a given miRNA (particle) was detected using a Guava easyCyte 8HT flow cytometer. Additionally, miR-19b-3p, Let-7i-5p and Let-7b-5p were included as endogenous controls, as they demonstrated the least variation across all samples in our sequencing results. Raw data were processed using FireCode software (Firefly BioWorks), and normalized to the geometric mean of the endogenous controls. We used a two-class paired significant analysis of microarray analysis to determine differential gene expression before and after treatment. *P*<0.05 was considered statistically significant.

Frozen plasma was thawed at room temperature, and 625 μl of each sample was used for total RNA extraction using the mirVana Isolation kit (ThermoFisher), following the manufacturer's instructions. Libraries were prepared using the Ion PI Template OT2 200 kit Version 3 and sequenced on an Ion Proton System (ThermoFisher). FASTQ files were downloaded from Ion Suite software and aligned using miRBase (V20) and the hg19 genome build.

Gene targets of miR-146a-5p, miR-146b-5p, miR-24–3p and miR-425-3p were identified using seven miRNA target prediction databases: miRWalk 2.0, miRDB, miRanda, RNA22 v2, RNAHybrid, TargetScan and DIANA Tools^42^,^43^,^44^,^45^,^46^,^47^,^48^. We then used DAVID v6.7 to identify potential biological pathways regulated by these miRNAs. To validate our miRNA target prediction and pathway analysis, we performed a second, independent, *in silico* analysis for miR-146a-5p, miR-146b-5p, miR-24-3p and miR-425-3p using DIANA: miRPath. This program allows identification of common miRNA targets provided by the DIANA-microT-CDS algorithm derived from DIANA-TarBase v6.0. In addition, miRPath can perform a pathway analysis based on various levels of miRNA interactions.

For genome-wide expression analysis in peripheral blood samples, we used the Human HT-12 v4 Expression Bead Chip (Illumina). Background adjustment using variance-stabilizing transformation correction and quantile normalization was performed using the FlexArray package implemented in R (version 1.6.3). Probes with >70% absent points among all samples were removed from downstream analysis. A total of ∼15,000 probes were included in the final analysis. Microarray data were analysed using a two-class paired significant analysis of microarray, as well as Pearson's correlations to determine differential gene expression before and after treatment using or correlations between miRNAs and target genes, respectively. All analyses were performed using MultiExperiment Viewer 4 (MeV4, TM4 software suite). *P*<0.05 was considered statistically significant.

For microRNA quantification, total RNA samples were reverse transcribed and quantified using TaqMan RT-PCR microRNA assays (Applied Biosystems) according to the manufacturer's instructions. Real-time PCR reactions were run in triplicate using the ABI 7900HT Fast Real-Time PCR System and data were collected using the Sequence Detection System 2.4 (SDS) software (Applied Biosystems). To measure miRNA expression, we used miRNA TaqMan probes, considered to be the gold standard for miRNA quantification^5^. Expression was calculated using the absolute quantitation standard curve method. RNU6B was used as an endogenous control as it showed expression levels that remained relatively constant with low variance and high abundance across the samples tested. All primers used are described in Supplementary Table 15. All numerical data are expressed as the mean±s.e.m. Statistical differences among groups were analysed by Student's *t*-test. Statistical significance was calculated using GraphPad Prism5 and SPSS 20. *P*<0.05 was considered statistically significant. For messenger RNA quantification, total RNA was reverse transcribed using MMLV reverse transcriptase (Gibco) and oligo(dT)16 primers (Invitrogen). Real-time PCR reactions were run in triplicate using the ABI QuantStudio 6 Flex Real-Time PCR System and data were collected using the QuantStudio Real-Time PCR software (Applied Biosystems). Expression levels were calculated using the relative quantification method. Both *β-actin* and *GAPDH* were used as endogenous controls. All primers can be found in Supplementary Table 15.

Gene expression data have been deposited in the Gene Expression Omnibus (GEO) database under the accession code GSE97154 (https://www.ncbi.nlm.nih.gov/geo/query/acc.cgi?acc=GSE97154).

### Animal experiments

Male 10-week-old C57BL/6J mice, and 6-month-old CD1 retired breeders, were maintained on a 12 h light–dark cycle (lights on at 0700, h) at 22–25 °C with *ad libitum* access to food and water. C57 mice were housed 5 per cage except following defeat experiments at which point mice were singly housed. All behavioural testing occurred during the animals' light cycle. The experimenter was blinded to the experimental group and the order of testing was counterbalanced during behavioural experiments. All experiments were conducted in accordance with the guidelines of the Institutional Animal Care and Use Committee at Icahn School of Medicine at Mount Sinai.

C57BL/6J mice were subjected to CSDS for 10 consecutive days, as described previously^49^,^50^. Briefly, C57BL/6J mice were subjected to 10 daily, 5 min defeats by a novel CD1 aggressor mouse and were then housed across a plexiglass divider to allow for sensory contact. Control mice were housed in cages separated from other control mice by a plexiglass divider and were rotated to a different cage daily. Resilient and susceptible mice were identified by their respective preference for, or avoidance of, interaction with a novel mouse after 10 days of defeat in a social interaction test. Only control and susceptible mice were used for further study with imipramine.

Social-avoidance behaviour was assessed with a novel CD1 mouse in a two-stage social-interaction test. In the first 2.5 min test (no target), the experimental mouse was allowed to freely explore an arena (44 × 44 cm) containing a plexiglass and wire mesh enclosure (10 × 6 cm) against one wall of the arena. In the second 2.5 min test (target), the experimental mouse was returned to the arena with a novel CD1 mouse enclosed in the plexiglass wire mesh cage. Time spent in the ‘interaction zone' (14 × 26 cm) surrounding the plexiglass wire mesh cage, ‘corner zones' (10 × 10 cm) and ‘distance travelled' within the arena was measured by video tracking software (Ethovision 5.0, Noldus).

Sucrose preference was assessed using a 2% sucrose solution or tap water in 50 ml tubes with stoppers fitted with ball-point sipper tubes. All animals were acclimatized to two-bottle choice conditions before testing conditions. Daily, at ∼1600, h, fluid levels were noted, and tube positions were interchanged. Sucrose preference was calculated as a percentage (100 × volume of sucrose consumed/total volume consumed (sucrose and water)) and was averaged over 2 days of testing.

Susceptible mice were randomly assigned to treatment with either imipramine (20 mg per kg, daily) or a saline control (daily) treatment for 14 days. Blood was collected from all animals via the saphenous vein before treatment. Rapid sampling from the saphenous vein was used because it causes minimal discomfort or stress to the animal. The blood was stored in RNAlater at −70 °C until use. After treatment and the second round of behaviour testing, animals were killed and blood was collected via truncation into RNAlater and frozen at −70 °C until use.

Then, 100 ng of total RNA was used to quantify expression levels of miRNAs from animal blood samples using a probe-based miRNA expression assay (NanoString Technologies, USA). Briefly, hybridization of specific tags to target miRNAs was annealed to create multiplex probe libraries from all samples. Each miRNA of interest is identified by a ‘colour code' generated by six ordered fluorescent spots present on a reporter probe and read by the nCounter Prep Station Digital Analyzer. Control RNA was included to monitor ligation efficiency and specificity throughout each step of the reactions. In addition, this protocol does not include any amplification steps that might introduce bias to the results. Raw data were imported into NSolver Analysis Software v2.0 (NanoString technologies) and normalized to the average counts to all endogenous genes. Data were further normalized using internal positive spike-in controls to account for variability in the hybridization process. Normalized data were imported into MultiExperiment Viewer (MeV) software v.4.9 for further analysis. All numerical data are expressed as the mean±s.e.m. Statistical differences among groups were analysed by Student's *t*-test. *P*<0.05 was considered statistically significant.

### Cell culture

For miRNA Overexpression experiments, human embryonic kidney cells (HEK293) were cultured in Dulbecco's modified Eagle's medium supplemented with 10% fetal bovine serum, 100 U ml^−1^ penicillin and 100 μg ml^−1^ streptomycin (Invitrogen) in a 5% CO_2_ humidified incubator at 37 °C. For miRNA mimic treatments, cells were grown in the continuous presence of 5 nM miRNA Mimic (miR-146a-5p, miR-146b-5p, miR-24-3p or miR-425-3p), 5 nM miR-Mimic scramble control (AllStars Negative Control siRNA, Qiagen) or mock vehicle (HiPerFect Transfection Reagent, Qiagen) for 24 h after which cell pellets were collected and both mRNA and miRNA were extracted as previously explained. All experiments were performed in triplicate. All numerical data are expressed as the mean±s.e.m. Statistical differences among groups were analysed by one-way analysis of variance with *post hoc* correction and Pearson's correlation coefficients. Statistical significance was calculated using GraphPad Prism5 and SPSS 20. *P*<0.05 was considered statistically significant.

Human NPCs were treated as previously explained^12^. To find adequate antidepressant concentrations for treatment, cells were screened for cytotoxic effects by measuring the activity of mitochondrial dehydrogenase using the 3-(4, 5-dimethylthiazol-2-yl)-2, 5-diphenyltetrazolium bromide (MTT) assay (Sigma-Aldrich Co) and antidepressants were applied at concentrations that showed no toxicity in the MTT test. For chronic antidepressant treatment, cells were grown in the continuous presence of 10 μM duloxetine, or no-drug control for 15 days, after which cell pellets were collected and both mRNA and miRNA were extracted. All experiments were performed in triplicate. All numerical data are expressed as the mean±s.e.m. Statistical differences among groups were analysed by independent Student's *t*-tests. Statistical significance was calculated using GraphPad Prism5 and SPSS 20. *P*<0.05 was considered statistically significant.

### Data availability

Gene expression data have been deposited in the GEO database under the accession code GSE97154. All other data that support the findings of this study are available from the corresponding author on reasonable request (https://www.ncbi.nlm.nih.gov/geo/query/acc.cgi?acc=GSE97154).

## Additional information

**How to cite this article:** Lopez, J. P. *et al*. MicroRNAs 146a/b-5p and 425-3p and 24-3p are markers of antidepressant response and regulate MAPK/Wnt-system genes. *Nat. Commun.* **8,** 15497 doi: 10.1038/ncomms15497 (2017).

**Publisher's note:** Springer Nature remains neutral with regard to jurisdictional claims in published maps and institutional affiliations.

## Supplementary Material

Supplementary Information Supplementary Figures, Supplementary Tables and Supplementary References

## Figures and Tables

**Table 1:** Duloxetine responders.

**hsa-miRNA**	**FC (T0 versus T8)**	**Adjusted** ***P*** **value**
miR-425-3p	0.46	2.639E−08
miR-24-3p	0.70	6.813E−06
miR-503-5p	0.45	6.987E−06
miR-146a-5p	0.60	3.748E−05
miR-215-5p	1.50	8.744E−05
miR-3074-5p	0.76	1.616E−04
miR-1180-3p	0.75	2.487E−04
miR-425-5p	0.86	0.001
miR-324-5p	0.78	0.001
miR-146b-5p	0.66	0.001
miR-6750-3p	1.27	0.005
miR-6511a-3p	1.21	0.007
miR-361-5p	0.84	0.009
miR-3173-5p	1.30	0.023
miR-2110	0.81	0.026
miR-3605-3p	1.17	0.032
miR-6881-3p	1.27	0.032
miR-30e-5p	1.19	0.035
miR-423-3p	1.12	0.035
miR-361-3p	1.10	0.040
miR-3184-5p	1.12	0.050
hsa-miR-636	1.14	0.050

FC, fold change; hsa, *Homo sapiens*.

Fold change is reported in the centre column and miRNAs are ranked according to adjusted *P* values (Benjamini–Hochberg correction for multiple testing).

**Table 2:** Placebo responders.

**hsa-miRNA**	**FC (T0 versus T8)**	**Adjusted** ***P*** **value**
miR-146a-5p	0.49	1.860E−05
miR-425-3p	0.50	1.415E−04
miR-24-3p	0.76	3.255E−04
miR-146b-5p	0.79	0.004
miR-503-5p	0.48	0.007
miR-3074-5p	0.73	0.007

FC, fold change; hsa, *Homo sapiens*.

Fold change is reported in the centre column and miRNAs are ranked according to adjusted *P* values (Benjamini–Hochberg correction for multiple testing).

**Table 3:** Animal model of MDD.

**mmu-miRNA**	**Controls saline treated**	**Susceptible saline treated**	**Susceptible imipramine (RES)**	**Susceptible imipramine (NRES)**
miR-425	0.956	0.493	0.007	0.618
miR-146b	0.653	0.557	0.041	0.304
miR-24	0.823	0.592	0.047	0.240
miR-146a	0.939	0.392	0.090	0.213

NRES, nonresponders; RES, responders.

*P* values across treatment groups.

**Table 4:** MiRNA expression in vPFC of post-mortem brains of subjects with MDD and controls.

**hsa-miRNA**	**FC**	***P*** **value**
miR-425-3p	1.31	0.005
miR-24-3p	1.26	0.037
miR-146a-5p	1.28	0.031
miR-146b-5p	1.29	0.028

FC, fold change; hsa, *Homo sapiens*.

Quantitative real-time (qRT-PCR) expression data in vPFC of post-mortem brains of subjects with MDD and controls.

## References

[b1] Schmidt H. D., Shelton R. C. & Duman R. S. Functional biomarkers of depression: diagnosis, treatment, and pathophysiology. Neuropsychopharmacology 36, 2375–2394 (2011). 21814182 10.1038/npp.2011.151 PMC3194084

[b2] Banthin J. S. & Miller G. E. Trends in prescription drug expenditures by Medicaid enrollees. Med. Care 44, I27–I35 (2006). 16625061 10.1097/01.mlr.0000208132.36055.84

[b3] Chen Y. . Utilization, price, and spending trends for antidepressants in the US Medicaid Program. Res. Social Adm. Pharm. 4, 244–257 (2008). 18794035 10.1016/j.sapharm.2007.06.019

[b4] Esteller M. Non-coding RNAs in human disease. Nat. Rev. Genet. 12, 861–874 (2011). 22094949 10.1038/nrg3074

[b5] Lopez J. P. . Biomarker discovery: quantification of microRNAs and other small non-coding RNAs using next generation sequencing. BMC Med. Genomics 8, 35 (2015). 26130076 10.1186/s12920-015-0109-x PMC4487992

[b6] He L. & Hannon G. J. MicroRNAs: small RNAs with a big role in gene regulation. Nat. Rev. Genetics 5, 522–531 (2004). 15211354 10.1038/nrg1379

[b7] Qureshi I. A. & Mehler M. F. Emerging roles of non-coding RNAs in brain evolution, development, plasticity and disease. Nat. Rev. Neurosci. 13, 528–541 (2012). 22814587 10.1038/nrn3234 PMC3478095

[b8] Baudry A., Mouillet-Richard S., Schneider B., Launay J. M. & Kellermann O. miR-16 targets the serotonin transporter: a new facet for adaptive responses to antidepressants. Science 329, 1537–1541 (2010). 20847275 10.1126/science.1193692

[b9] Launay J. M., Mouillet-Richard S., Baudry A., Pietri M. & Kellermann O. Raphe-mediated signals control the hippocampal response to SRI antidepressants via miR-16. Transl. Psychiatry 1, e56 (2011). 22833211 10.1038/tp.2011.54 PMC3309472

[b10] Issler O. . MicroRNA 135 is essential for chronic stress resiliency, antidepressant efficacy, and intact serotonergic activity. Neuron 83, 344–360 (2014). 24952960 10.1016/j.neuron.2014.05.042

[b11] Dias C. . beta-catenin mediates stress resilience through Dicer1/microRNA regulation. Nature 516, 51–55 (2014). 25383518 10.1038/nature13976 PMC4257892

[b12] Lopez J. P. . miR-1202 is a primate-specific and brain-enriched microRNA involved in major depression and antidepressant treatment. Nat. Med. 20, 764–768 (2014). 24908571 10.1038/nm.3582 PMC4087015

[b13] Kennedy S. H., Andersen H. F. & Lam R. W. Efficacy of escitalopram in the treatment of major depressive disorder compared with conventional selective serotonin reuptake inhibitors and venlafaxine XR: a meta-analysis. J. Psychiatry Neurosci. 31, 122–131 (2006). 16575428 PMC1413963

[b14] Kennedy S. H. . The Canadian Biomarker Integration Network in Depression (CAN-BIND): advances in response prediction. Curr. Pharm. Des. 18, 5976–5989 (2012). 22681173 10.2174/138161212803523635

[b15] Kennedy S. H., Lam R. W., Parikh S. V., Patten S. B. & Ravindran A. V. Canadian Network for Mood and Anxiety Treatments (CANMAT) clinical guidelines for the management of major depressive disorder in adults. Introduction. J. Affect. Disord. 117, S1–S2 (2009). 19682750 10.1016/j.jad.2009.06.043

[b16] Pecina M. . Association between placebo-activated neural systems and antidepressant responses: neurochemistry of placebo effects in major depression. JAMA Psychiatry 72, 1087–1094 (2015). 26421634 10.1001/jamapsychiatry.2015.1335 PMC4758856

[b17] Pecina M. & Zubieta J. K. Molecular mechanisms of placebo responses in humans. Mol. Psychiatry 20, 416–423 (2015). 25510510 10.1038/mp.2014.164 PMC4372496

[b18] Uher R. . Differential efficacy of escitalopram and nortriptyline on dimensional measures of depression. Br. J. Psychiatry 194, 252–259 (2009). 19252156 10.1192/bjp.bp.108.057554

[b19] Lam R. W. . Discovering biomarkers for antidepressant response: protocol from the Canadian biomarker integration network in depression (CAN-BIND) and clinical characteristics of the first patient cohort. BMC Psychiatry 16, 105 (2016). 27084692 10.1186/s12888-016-0785-x PMC4833905

[b20] Krishnan V. . Molecular adaptations underlying susceptibility and resistance to social defeat in brain reward regions. Cell 131, 391–404 (2007). 17956738 10.1016/j.cell.2007.09.018

[b21] Huang, da W., Sherman B. T. & Lempicki R. A. Systematic and integrative analysis of large gene lists using DAVID bioinformatics resources. Nat. Protoc. 4, 44–57 (2009). 19131956 10.1038/nprot.2008.211

[b22] Vlachos I. S. . DIANA-miRPath v3.0: deciphering microRNA function with experimental support. Nucleic Acids Res. 43, W460–W466 (2015). 25977294 10.1093/nar/gkv403 PMC4489228

[b23] Duman R. S., Li N., Liu R. J., Duric V. & Aghajanian G. Signaling pathways underlying the rapid antidepressant actions of ketamine. Neuropharmacology 62, 35–41 (2012). 21907221 10.1016/j.neuropharm.2011.08.044 PMC3195863

[b24] Perroud N. . Genetic predictors of increase in suicidal ideation during antidepressant treatment in the GENDEP project. Neuropsychopharmacology 34, 2517–2528 (2009). 19641488 10.1038/npp.2009.81

[b25] Perroud N. . Genome-wide association study of increasing suicidal ideation during antidepressant treatment in the GENDEP project. Pharmacogenomics J. 12, 68–77 (2012). 20877300 10.1038/tpj.2010.70

[b26] Duric V. & Duman R. S. Depression and treatment response: dynamic interplay of signaling pathways and altered neural processes. Cell. Mol. Life Sci. 70, 39–53 (2013). 22585060 10.1007/s00018-012-1020-7 PMC3952234

[b27] Yi Z. . Blood-based gene expression profiles models for classification of subsyndromal symptomatic depression and major depressive disorder. PLoS ONE 7, e31283 (2012). 22348066 10.1371/journal.pone.0031283 PMC3278427

[b28] Hepgul N., Cattaneo A., Zunszain P. A. & Pariante C. M. Depression pathogenesis and treatment: what can we learn from blood mRNA expression? BMC Med. 11, 28 (2013). 23384232 10.1186/1741-7015-11-28 PMC3606439

[b29] Jansen R. . Gene expression in major depressive disorder. Mol. Psychiatry 21, 444 (2016). 26100536 10.1038/mp.2015.94

[b30] Xu F. . Differential co-expression and regulation analyses reveal different mechanisms underlying major depressive disorder and subsyndromal symptomatic depression. BMC Bioinformatics 16, 112 (2015). 25880836 10.1186/s12859-015-0543-y PMC4434877

[b31] Chao M. V. Neurotrophins and their receptors: a convergence point for many signalling pathways. Nat. Rev. Neurosci. 4, 299–309 (2003). 12671646 10.1038/nrn1078

[b32] Lopez J. P. . Epigenetic regulation of BDNF expression according to antidepressant response. Mol. Psychiatry 18, 398–399 (2012). 22547115 10.1038/mp.2012.38 PMC5319860

[b33] Turner C. A., Akil H., Watson S. J. & Evans S. J. The fibroblast growth factor system and mood disorders. Biol. Psychiatry 59, 1128–1135 (2006). 16631131 10.1016/j.biopsych.2006.02.026

[b34] Illumina. Illumina CASAVA 1.8 http://support.illumina.com/content/dam/illumina-support/documents/myillumina/33d66b02-53b5-4f4d-9d8b-f94237c7e44d/casava_qrg_15011197b.pdf (2011).

[b35] Song L., Florea L. & Langmead B. Lighter: fast and memory-efficient sequencing error correction without counting. Genome Biol. 15, 509 (2014). 25398208 10.1186/s13059-014-0509-9 PMC4248469

[b36] Kent W. J. . The human genome browser at UCSC. Genome Res. 12, 996–1006 (2002). 12045153 10.1101/gr.229102 PMC186604

[b37] Chen C. J. . ncPRO-seq: a tool for annotation and profiling of ncRNAs in sRNA-seq data. Bioinformatics 28, 3147–3149 (2012). 23044543 10.1093/bioinformatics/bts587

[b38] Kozomara A. & Griffiths-Jones S. miRBase: integrating microRNA annotation and deep-sequencing data. Nucleic Acids Res. 39, D152–D157 (2011). 21037258 10.1093/nar/gkq1027 PMC3013655

[b39] Kozomara A. & Griffiths-Jones S. miRBase: annotating high confidence microRNAs using deep sequencing data. Nucleic Acids Res. 42, D68–D73 (2014). 24275495 10.1093/nar/gkt1181 PMC3965103

[b40] Love M. I., Huber W. & Anders S. Moderated estimation of fold change and dispersion for RNA-seq data with DESeq2. Genome Biol. 15, 550 (2014). 25516281 10.1186/s13059-014-0550-8 PMC4302049

[b41] Li J. & Tibshirani R. Finding consistent patterns: a nonparametric approach for identifying differential expression in RNA-Seq data. Stat. Methods Med. Res. 22, 519–536 (2013). 22127579 10.1177/0962280211428386 PMC4605138

[b42] Dweep H. & Gretz N. miRWalk2.0: a comprehensive atlas of microRNA-target interactions. Nat. Methods 12, 697 (2015). 26226356 10.1038/nmeth.3485

[b43] Wong N. & Wang X. miRDB: an online resource for microRNA target prediction and functional annotations. Nucleic Acids Res. 43, D146–D152 (2015). 25378301 10.1093/nar/gku1104 PMC4383922

[b44] Betel D., Koppal A., Agius P., Sander C. & Leslie C. Comprehensive modeling of microRNA targets predicts functional non-conserved and non-canonical sites. Genome Biol. 11, R90 (2010). 20799968 10.1186/gb-2010-11-8-r90 PMC2945792

[b45] Miranda K. C. . A pattern-based method for the identification of MicroRNA binding sites and their corresponding heteroduplexes. Cell 126, 1203–1217 (2006). 16990141 10.1016/j.cell.2006.07.031

[b46] Kruger J. & Rehmsmeier M. RNAhybrid: microRNA target prediction easy, fast and flexible. Nucleic Acids Res. 34, W451–W454 (2006). 16845047 10.1093/nar/gkl243 PMC1538877

[b47] Agarwal V., Bell G. W., Nam J. W. & Bartel D. P. Predicting effective microRNA target sites in mammalian mRNAs. eLife 4, e05005 (2015). 10.7554/eLife.05005 PMC4532895 26267216

[b48] Paraskevopoulou M. D. . DIANA-microT web server v5.0: service integration into miRNA functional analysis workflows. Nucleic Acids Res. 41, W169–W173 (2013). 23680784 10.1093/nar/gkt393 PMC3692048

[b49] Golden S. A., Covington H. E. 3rd, Berton O. & Russo S. J. A standardized protocol for repeated social defeat stress in mice. Nat. Protoc. 6, 1183–1191 (2011). 21799487 10.1038/nprot.2011.361 PMC3220278

[b50] Berton O. & Nestler E. J. New approaches to antidepressant drug discovery: beyond monoamines. Nat. Rev. Neurosci. 7, 137–151 (2006). 16429123 10.1038/nrn1846

