# Supplementary material for: MicroRNAs 146a/b-5 and 425-3p and 24-3p are markers of antidepressant response and regulate MAPK/Wnt-system genes
Source: Nat Commun. 2017 May 22;8:15497. doi: 10.1038/ncomms15497 (PMC5477510; doi:10.1038/ncomms15497)
Supplement: Supplementary Information — Supplementary Figures, Supplementary Tables and Supplementary References [file ncomms15497-s1.pdf]

SUPPLEMENTARY FIGURES

Supplementary Figure. 1. Validation in Plasma: Expression (number of reads/million, Log2) levels of miR-146a-5p, miR-146b-5p, and miR-24-3p after 8 weeks in MDD patients who responded to duloxetine treatment. ***indicates P<0.001. Levels of miRNA in plasma are considerably lower and more variable than in whole blood. We were not able to obtain reliable measures for miR-425-3p in plasma samples.

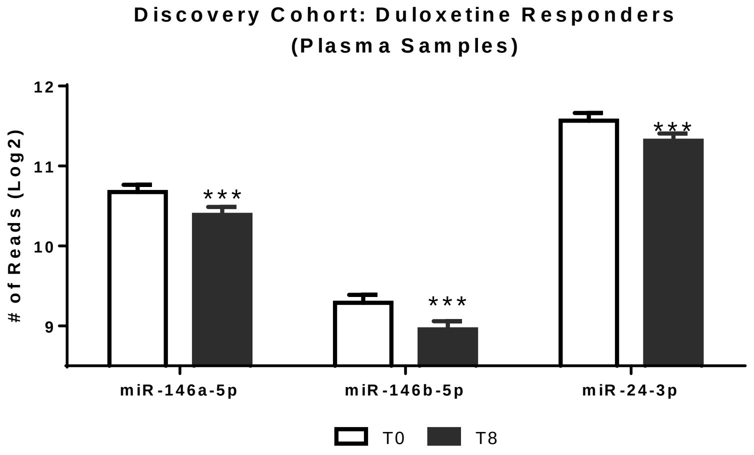

Supplementary Figure. 2. MicroRNA correlations in brain: Scatterplots of Pearson correlations between differentially expressed miRNAs in postmortem brain (vPFC) samples.

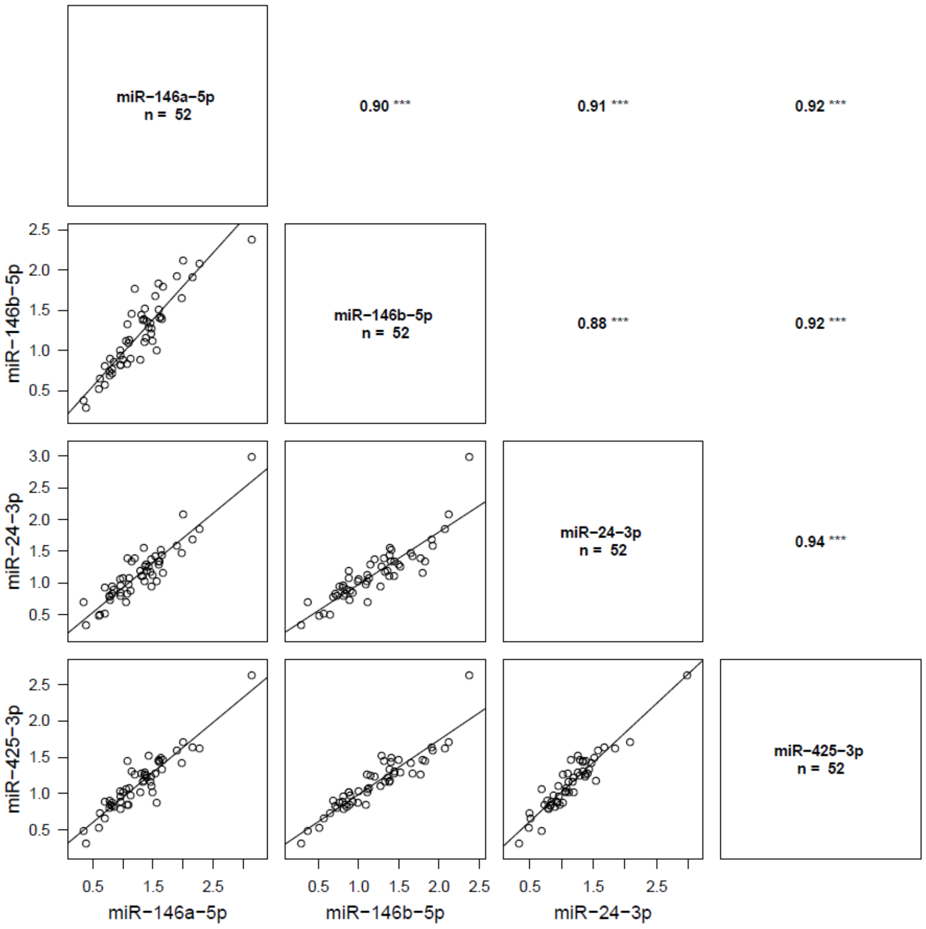

Supplementary Figure. 3. Discovery Cohort – Predicted target genes involved in MAPK and Wnt signaling pathways: Differential expression analysis of MAPK/Wnt genes identified through our computational analysis. Most of the predicted targets were significantly dysregulated after duloxetine treatment (25 out of 36 genes, 69%).

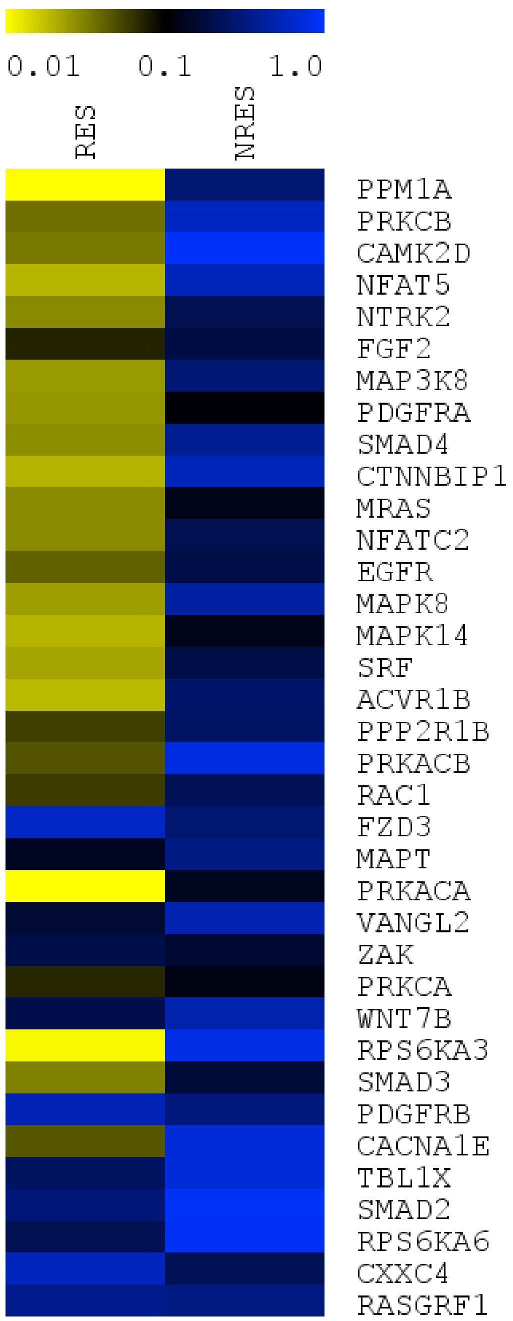

Supplementary Figure. 4. Expression of miR-146a-5p, miR-146b-5p, miR-24-3p and miR-425-3p in human whole-blood and postmortem brain samples: Small RNA sequencing data (number of reads/million, Log2) expression data for miR-146a-5p, miR-146b-5p, miR-24-3p and miR-425-3p in whole-blood and vPFC of postmortem brains.

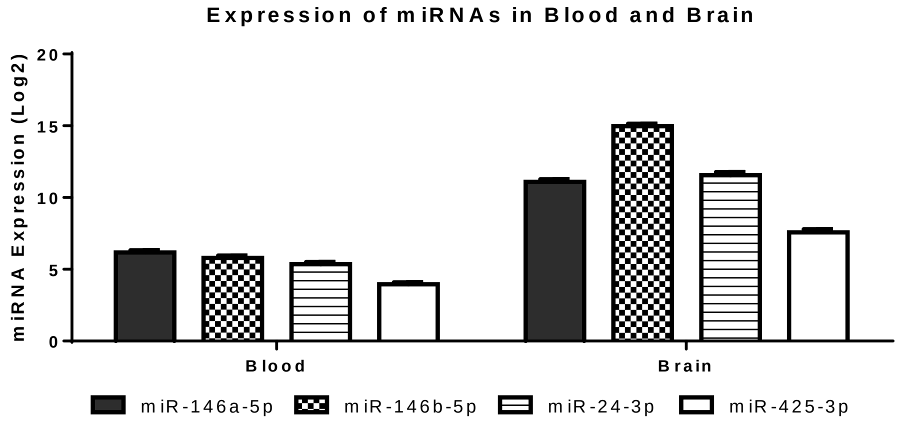

Supplementary Figure. 5. Expression of miR-146a-5p, miR-146b-5p, miR-24-3p and miR-425-3p in other human biological fluids: Small RNA sequencing data (number of reads/million, Log2) expression data for miR-146a-5p, miR-146b-5p, miR-24-3p and miR-425-3p in human plasma (N=37), serum (N=13), saliva (N=3) and semen/sperm (N=4). miRmine Database: http://guanlab.ccmb.med.umich.edu/mirmine/

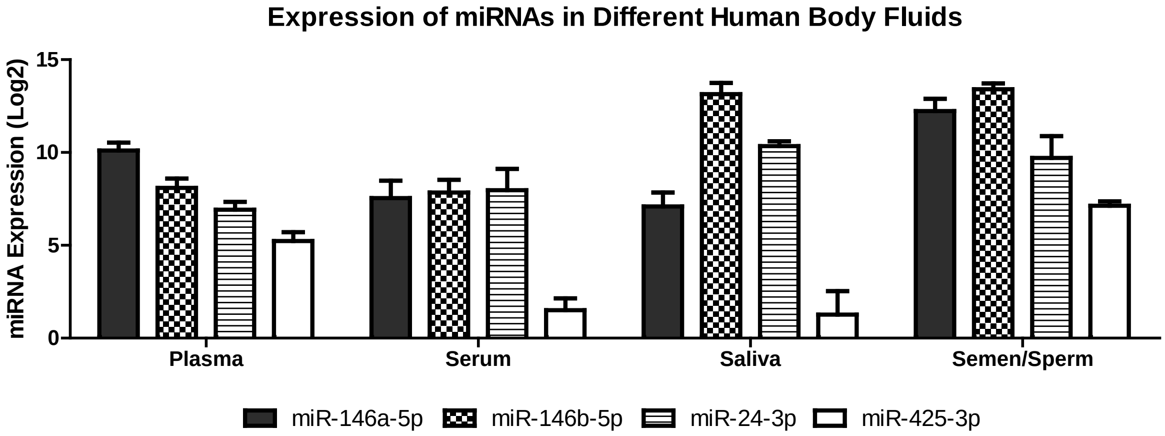

SUPPLEMENTARY TABLES

Supplementary Table 1. Duloxetine Treated Samples: Expression of miR-146a-5p, miR-146b-5p, miR-24-3p, and miR-425-3p in responders vs. non-responders from the discovery cohort.

microRNA	Adj P value - Responders	Adj P value – Non-Responders
hsa-miR-425-3p	2.63949E-08	0.205673072
hsa-miR-24-3p	6.81342E-06	0.271682804
hsa-miR-146a-5p	3.74787E-05	0.271682804
hsa-miR-146b-5p	0.001448493	0.722733069

Supplementary Table 2. Placebo Treated Samples: Expression of miR-146a-5p, miR-146b-5p, miR-24-3p, and miR-425-3p in responders vs. non-responders from the discovery cohort.

microRNA	Adj P value - Responders	Adj P value – Non-Responders
hsa-miR-425-3p	0.000141454	0.278461101
hsa-miR-24-3p	0.000325475	0.203927569
hsa-miR-146a-5p	1.86011E-05	0.269224663
hsa-miR-146b-5p	0.003720756	0.59572429

Supplementary Table 3. Technical Validation: MicroRNAs selected for technical validation. In total, 13 out 15 (87%) miRNA targets tested were validated. Small RNA sequencing results were validated with a custom miRNA panel using circulatory miRNA assays by Firefly (BioWorks). Bold font denotes statistical significance.

miRNA	Group	FC	*P* value - RES
**miR-24-3p**	Target	**0,75**	**1,691E-07**
**miR-425-3p**	Target	**0,62**	**5,373E-04**
miR-324-5p	Target	0,77	**1,366E-03**
miR-361-3p	Target	0,78	**0,002**
miR-425-5p	Target	0,83	**0,002**
miR-423-3p	Target	1,23	**0,004**
miR-361-5p	Target	0,81	**0,006**
**miR-146a-5p**	Target	**0,88**	**0,011**
miR-6750-3p	Target	1,41	**0,031**
**miR-146b-5p**	Target	**0,87**	**0,035**
miR-3173-5p	Target	1,20	**0,036**
miR-3605-3p	Target	1,22	**0,040**
miR-3074-5p	Target	0,69	**0,086**
miR-1180-3p	Target	1,03	0,417
miR-6511a-3p	Target	1,00	0,487
Let-7b-5p	End Control	0,99	0,462
Let-7i-5p	End Control	1,02	0,319
miR-19b-3p	End Control	0,97	0,311

Supplementary Table 4. Correlation between the five differentially expressed miRNAs in duloxetine and placebo responders. Significant *P* values and Pearson coefficients (r).

Pearson's r / (*P* Values)	miR-146a-5p	miR-146b-5p	miR-24-3p	miR-3074-5p	miR-425-3p
**miR-146a-5p**	1	X	X	X	X
**miR-146b-5p**	0.70 (6.9E-38)	1	X	X	X
**miR-24-3p**	0.70 (8.5E-38)	0.39 (3.1E-10)	1	X	X
**miR-3074-5p**	0.62 (3.2E-27)	0.29 (3.1E-6)	0.97 (1.2E-162)	1	X
**miR-425-3p**	0.77 (1.6E-50)	0.55 (2.8E-21)	0.73 (6.8E-43)	0.64 (2.1E-30)	1

Supplementary Table 5. Replication Cohort 1. Differential expression of miRNAs after 8 weeks of antidepressant treatment. MiRNAs are ranked according to *P* values and fold change (FC).

miRNA	FC (T0 vs T8)	P value - RES
**miR-425-3p**	0.81	0.023
**miR-24-3p**	0.83	0.035
**miR-146a-5p**	0.82	0.030
**miR-146b-5p**	0.83	0.041
**miR-3074-5p**	**0.95**	**0.333**

Supplementary Table 6. Replication Cohort 2. Differential expression of miRNAs after 8 weeks of antidepressant treatment. MiRNAs are ranked according to *P* values and fold change (FC).

miRNA	FC (T0 vs T8)	P value - RES
**miR-425-3p**	0.99	0.480
**miR-24-3p**	0.89	0.023
**miR-146a-5p**	0.80	0.045
**miR-146b-5p**	0.73	0.030

Supplementary Table 7. Hematology data analysis: Cell count analysis performed in blood samples from MDD patients treated with Duloxetine (Discovery Cohort).

	Responders -Duloxetine			Non-Responders – Duloxetine		
**Cell Type**	**Baseline**	**After Treatment**	**P-value**	**Baseline**	**After Treatment**	**P-value**
**Leukocytes**	6.5 ± 1.6	6.4 ± 1.4	**0,76**	6.69 ± 1.9	6.9 ± 2.1	**0,25**
**Basophils**	0.35 ± 0.19	0.33 ± 0.2	**0,84**	0.36 ± 0.19	0.37 ± 0.2	**0,55**
**Eosinophils**	2.3 ± 0.16	2.5 ± 0.16	**0,11**	2.9 ± 0.21	2.9 ± 0.22	**0,45**
**Lymphocytes**	29.2 ± 6.8	29.5 ± 6.8	**0,95**	32.4 ± 9.2	30.1 ± 7.3	**0,41**
**Monocytes**	6.6 ± 2.3	6.7 ± 2.0	**0,51**	6.5 ± 2.0	6.7 ± 2.2	**0,64**
**Neutrophils**	61.6 ± 8.6	60.7 ± 8.3	**0,57**	57.9 ± 10.8	58.6 ± 8.4	**0,80**

Supplementary Table 8. Hematology data analysis: Cell count analysis performed in blood samples from MDD patients treated with a placebo control (Discovery Cohort).

	Responders -Placebo			Non-Responders – Placebo		
**Cell Type**	**Baseline**	**After Treatment**	**P-value**	**Baseline**	**After Treatment**	**P-value**
**Leukocytes**	6.6 ± 2.1	6.5 ± 1.5	**0,67**	6.8 ± 1.9	6.7 ± 1.9	**0,61**
**Basophils**	0.44 ± 0.3	0.43 ± 0.2	**0,86**	0.35 ± 0.2	0.41 ± 0.2	**0,24**
**Eosinophils**	2.3 ± 0.17	2.9 ± 0.2	**0,34**	2.5 ± 0.14	2.5 ± 0.177	**0,57**
**Lymphocytes**	30.2 ± 8.6	31.0 ± 7.0	**0,62**	31.1 ± 8.2	31.4 ± 6.9	**0,70**
**Monocytes**	6.8 ± 2.9	7.2 ± 2.9	**0,37**	6.6 ± 2.4	6.1 ± 2.2	**0,28**
**Neutrophils**	59.1 ± 10.6	57.4 ± 9.4	**0,39**	59.5 ± 9.4	58.7 ± 7.7	**0,95**

Supplementary Table 9. Correlation between miR-146a-5p, miR-146b-5p, miR-24-3p and miR-425-3p in postmortem brains of subjects with MDD and controls. Significant P values and Pearson coefficients (r).

**Pearson's r / (*P* Values)**	**miR-146a-5p**	**miR-146b-5p**	**miR-24-3p**	**miR-425-3p**
**miR-146a-5p**	1	X	X	X
**miR-146b-5p**	0.91 (9.2E-20)	1	X	X
**miR-24-3p**	0.91 (2.8E-20)	0.88 (7.0E-18)	1	X
**miR-425-3p**	0.92 (9.9E-22)	0.92 (6.2E-22)	0.94 (2.5E-24)	1

Supplementary Table 10. Discovery Cohort – Predicted target genes involved in MAPK and Wnt signaling pathways: Correlation analysis between the expression of miR-146a-5p, miR-146b-5p, miR-24-3p and miR-425-3p with genes identified through our computational analysis. Most of the predicted targets were significantly correlated with one or more of the miRNAs tested (32 out of 36 genes, 89%).

Gene Symbol	# of Pathways	Signaling Pathway	Correlated with miRNAs
**CACNA1E**	2	MAPK	**Yes**
**MAPT**	1	MAPK	**Yes**
**NTRK2**	1	MAPK	**Yes**
**PDGFRB**	2	MAPK	**Yes**
**PPM1A**	1	MAPK	**Yes**
**SRF**	1	MAPK	**Yes**
**PRKACA**	3	MAPK, Wnt	**Yes**
**PRKCA**	3	MAPK, Wnt	**Yes**
**PRKCB**	3	MAPK, Wnt	**Yes**
**RAC1**	3	MAPK, Wnt	**Yes**
**CTNNBIP1**	1	Wnt	**Yes**
**NFAT5**	1	Wnt	**Yes**
**PPP2R1B**	1	Wnt	**Yes**
**SMAD3**	2	Wnt	**Yes**
**RPS6KA3**	1	MAPK	**Yes**
**MAPK8**	2	MAPK, Wnt	**Yes**
**PRKACB**	3	MAPK, Wnt	**Yes**
**CAMK2D**	2	Wnt	**Yes**
**TBL1X**	1	Wnt	**Yes**
**WNT7B**	1	Wnt	**Yes**
**ACVR1B**	3	MAPK	**Yes**
**EGFR**	4	MAPK	**Yes**
**FGF2**	1	MAPK	**Yes**
**MAP3K8**	1	MAPK	**Yes**
**MRAS**	1	MAPK	**Yes**
**PDGFRA**	3	MAPK	**Yes**
**ZAK**	1	MAPK	**Yes**
**NFATC2**	2	MAPK, Wnt	**Yes**
**FZD3**	1	Wnt	**Yes**
**SMAD4**	2	Wnt	**Yes**
**VANGL2**	1	Wnt	**Yes**
**MAPK14**	1	MAPK	**Yes**
**RASGRF1**	1	MAPK	No
**RPS6KA6**	2	MAPK	No
**CXXC4**	1	Wnt	No
**SMAD2**	1	Wnt	No

Supplementary Table 11. Discovery Cohort – Predicted target genes involved in MAPK and Wnt signaling pathways: Differential expression analysis of MAPK/Wnt genes identified through our computational analysis. Most of the predicted targets were significantly dysregulated after duloxetine treatment (25 out of 36 genes, 69%).

	Responders				Non-Responders			
**Gene Name**	**T0 Mean**	**T8 Mean**	**FC**	**P value**	**T0 Mean**	**T8 Mean**	**FC**	**P value**
**PPM1A**	4.55	5.41	1.19	0.004420326	4.62	4.34	0.94	0.49965897
**PRKCB**	319.72	338.64	1.06	0.060316585	322.24	326.96	1.01	0.7897703
**CAMK2D**	2.61	2.82	1.08	0.057039768	2.69	2.69	1.00	0.98554325
**NFAT5**	6.50	7.31	1.12	0.03572427	7.75	7.50	0.97	0.7655441
**NTRK2**	2.36	2.52	1.07	0.051177796	2.44	2.33	0.95	0.3864045
**FGF2**	2.61	2.79	1.07	0.08750842	2.82	2.66	0.94	0.3462899
**MAP3K8**	45.66	49.45	1.08	0.045875274	38.48	43.25	1.12	0.5140519
**PDGFRA**	2.89	3.12	1.08	0.046876404	2.89	2.63	0.91	0.13049328
**SMAD4**	94.10	100.68	1.07	0.049906548	83.09	89.14	1.07	0.6193001
**CTNNBIP1**	8.75	10.30	1.18	0.036455154	12.65	11.97	0.95	0.7602985
**MRAS**	3.14	3.41	1.09	0.050751105	3.31	3.72	1.13	0.19830658
**NFATC2**	2.36	2.52	1.07	0.051177796	2.44	2.33	0.95	0.3864045
**EGFR**	2.61	2.80	1.07	0.06554178	2.17	2.26	1.04	0.3559935
**MAPK8**	3.12	3.42	1.08	0.044178944	3.28	3.41	1.04	0.6857121
**MAPK14**	5.04	5.74	1.14	0.03593131	6.52	5.15	0.79	0.19701816
**SRF**	182.04	191.19	1.05	0.041735854	178.86	185.74	1.04	0.3582986
**ACVR1B**	38.13	41.21	1.08	0.034682844	38.13	41.15	1.08	0.48104617
**PPP2R1B**	2.53	2.70	1.07	0.07770063	2.96	2.82	0.95	0.46333113
**PRKACB**	6.91	7.87	1.06	0.07025041	7.00	7.14	1.02	0.8925169
**RAC1**	503.27	522.19	1.04	0.07871647	507.27	493.01	0.97	0.41145188
**FZD3**	4.72	4.66	0.99	0.8001341	4.83	4.48	0.93	0.49779695
**MAPT**	4.66	4.32	0.93	0.22382466	3.99	4.15	1.04	0.5721705
**PRKACA**	2.31	2.54	1.10	0.002043212	2.28	2.48	1.09	0.21436845
**VANGL2**	3.20	3.04	0.95	0.28532743	3.11	3.22	1.03	0.7330913
**ZAK**	3.16	3.04	0.96	0.3574143	3.47	3.03	0.87	0.2767202
**PRKCA**	59.32	62.56	1.05	0.08641608	61.85	56.12	0.91	0.16824569
**WNT7B**	3.29	3.15	0.96	0.37274805	3.10	3.04	0.98	0.7147429
**RPS6KA3**	2.97	3.28	1.09	0.012417246	3.08	3.11	1.01	0.9133113
**SMAD3**	72.74	77.46	1.07	0.05446795	80.96	78.07	0.96	0.3105279
**PDGFRB**	3.55	3.61	1.02	0.7372547	4.35	4.11	0.95	0.53927463
**CACNA1E**	3.84	4.22	1.10	0.069353946	4.02	4.08	1.02	0.86569136
**TBL1X**	317.96	309.47	0.97	0.44723037	304.89	307.98	1.01	0.87157595
**SMAD2**	8.45	8.17	0.97	0.5262408	8.00	7.97	1.00	0.97446495
**RPS6KA6**	2.69	2.62	0.97	0.39195198	2.64	2.65	1.00	0.9641064
**CXXC4**	2.60	2.63	1.01	0.76980865	2.69	2.90	1.08	0.4009055
**RASGRF1**	2.79	2.85	1.02	0.5967571	2.76	2.66	0.96	0.5654304

Supplementary Table 12. Replication cohort – Predicted target genes involved in MAPK and Wnt signaling pathways: Correlation analysis between the expression of miR-146a-5p, miR-146b-5p, miR-24-3p and miR-425-3p with genes identified through our computational analysis. Most of the predicted targets were significantly correlated with one or more of the miRNAs tested (20 out of 28 genes, 71%).

Gene Symbol	# of Pathways	Signaling Pathway	Correlated with miRNAs
**PRKCA**	3	MAPK, Wnt	**Yes**
**PRKCB**	3	MAPK, Wnt	**Yes**
**RAC1**	3	MAPK, Wnt	**Yes**
**CTNNBIP1**	1	Wnt	**Yes**
**NFAT5**	1	Wnt	**Yes**
**SMAD3**	2	Wnt	**Yes**
**RPS6KA3**	1	MAPK	**Yes**
**PRKACB**	3	MAPK, Wnt	**Yes**
**CAMK2D**	2	Wnt	**Yes**
**ACVR1B**	3	MAPK	**Yes**
**EGFR**	4	MAPK	**Yes**
**MAPK14**	1	MAPK	**Yes**
**FZD3**	1	Wnt	**Yes**
**SMAD2**	1	Wnt	**Yes**
**MAPT**	1	MAPK	**Yes**
**NTRK2**	1	MAPK	**Yes**
**PPP2R1B**	1	Wnt	**Yes**
**ZAK**	1	MAPK	**Yes**
**CXXC4**	1	Wnt	**Yes**
**VANGL2**	1	Wnt	**Yes**
**PDGFRB**	2	MAPK	No
**SRF**	1	MAPK	No
**MRAS**	1	MAPK	No
**PPM1A**	1	MAPK	No
**PRKACA**	3	MAPK, Wnt	No
**TBL1X**	1	Wnt	No
**RASGRF1**	1	MAPK	No
**NFATC2**	2	MAPK, Wnt	No

Supplementary Table 13. Overexpression of miRNAs – Dysregulation of MAPK and Wnt genes: HEK293 cells were transfected for 24 hrs with a miRNA mimic for each of the individual miRNAs (miR-146a-5p, miR-146b-5p, miR-24-3p and miR-425-3p), a mock vehicle control or a miRNA-mimic scramble control. Results were compared to untreated control cells. All of the MAPK/Wnt genes tested were down-regulated by at least one of the miRNA mimics transfected. Bold font denotes statistical significance.

Gene	Untrs Ctrl	Vehicle Ctrl	Mimic Scramble	Mimic (miR-146a-5p)	Mimic (miR-146b-5p)	Mimic (miR-24-3p)	Mimic (miR-425-3p)
**NTRK2 (T1)**	1	0,9563	0,6539	**0,0308**	**0,0046**	**0,0044**	**0,0008**
**MAPK8**	1	0,1684	0,1195	**0,0052**	**0,0153**	**0,0305**	**0,0006**
**MAPT**	1	0,3775	0,2521	**0,0070**	**0,0012**	**0,0764**	**0,0173**
**PRKACA**	1	0,5554	0,4658	**0,0312**	**0,0183**	**0,0929**	**0,0094**
**PDGFRB**	1	0,1432	0,6751	**0,0287**	**0,0500**	**0,0530**	**0,0313**
**RPS6KA3**	1	0,5985	0,3422	**0,0284**	**0,0091**	**0,0137**	0,1300
**ZAK**	1	0,6916	0,2545	**0,0178**	**0,0122**	**0,0115**	0,1437
**PPM1A**	1	0,7750	0,2107	**0,0013**	**0,0091**	**0,0004**	0,1782
**FGF2**	1	0,6269	0,8467	**0,0039**	**0,0162**	**0,0187**	0,2939
**PDGFRA**	1	0,6373	0,3221	0,3271	**0,0370**	**0,0245**	**0,0347**
**MAPK14**	1	0,7948	0,5130	**0,0265**	**0,0335**	**0,0447**	0,3475
**PRKCA**	1	0,2576	0,4252	0,7020	**0,0037**	**0,0036**	**0,0277**
**PRKACB**	1	0,7682	0,2054	**0,0042**	0,8559	**0,0087**	**0,0403**
**SRF**	1	0,4667	0,4550	**0,0610**	**0,0111**	**0,0481**	**0,0630**
**RAC1**	1	0,9366	0,0897	**0,0581**	**0,0005**	**0,0994**	**0,0301**
**ACVR1B**	1	0,6816	0,3242	**0,0279**	0,1644	**0,0756**	**0,0079**
**EGFR**	1	0,8678	0,2799	**0,0126**	**0,0395**	**0,0972**	0,1635
**MAP3K8**	1	0,4425	0,8417	0,2709	**0,0226**	**0,0047**	**0,0733**
**RPS6KA6**	1	0,3079	0,7932	0,8192	**0,0102**	**0,0025**	**0,0835**
**NFATC2**	1	0,7783	0,4279	0,9689	**0,0305**	**0,0488**	0,1449
**PRKCB**	1	0,4853	0,7585	0,7473	0,6636	**0,0344**	**0,0312**
**MRAS**	1	0,2844	0,2957	**0,0865**	**0,0563**	0,5540	**0,0455**
**RASGRF1**	1	0,7801	0,1900	0,2000	0,2403	**0,0777**	0,3971
**B Actin**	1	0,6533	0,2446	0,5980	0,5678	0,3883	0,1525
**GAPDH**	1	0,5548	0,3238	0,3210	0,1964	0,7436	0,6392

Supplementary Table 14. MAPK and Wnt genes: Most of these genes have been previously associated with MDD or antidepressant activity (*supplementary references*).

#	Gene Symbol	Signaling Pathway	Literature References MDD or AD Treatment	Literature References Other Psychiatric Disorders
**1**	CACNA1E	MAPK	[1,2]	
**2**	MAPT	MAPK	[3]	
**3**	NTRK2	MAPK	[4,5,6]	
**4**	PDGFRB	MAPK	[7]	
**5**	PPM1A	MAPK	[8]	
**6**	SRF	MAPK	[9,1]	
**7**	PRKACA	MAPK, Wnt	[11]	
**8**	PRKCA	MAPK, Wnt	[12]	
**9**	PRKCB	MAPK, Wnt	[13,14]	
**10**	RAC1	MAPK, Wnt	[15]	
**11**	CTNNBIP1	Wnt	[16]	
**12**	NFAT5	Wnt	[17]	
**13**	PPP2R1B	Wnt	[18]	
**14**	SMAD3	Wnt	[19,2]	
**15**	RPS6KA3	MAPK	[21]	
**16**	MAPK8	MAPK, Wnt	[22]	
**17**	PRKACB	MAPK, Wnt	[11]	
**18**	CAMK2D	Wnt	[23]	
**19**	TBL1X	Wnt		
**20**	WNT7B	Wnt	[21]	
**21**	ACVR1B	MAPK	[7]	
**22**	EGFR	MAPK	[24,25]	
**23**	FGF2	MAPK	[26,27]	
**24**	MAP3K8	MAPK		[28]
**25**	MAPK14	MAPK		[29]
**26**	MRAS	MAPK		
**27**	PDGFRA	MAPK	[30,31]	
**28**	RASGRF1	MAPK	[32,33]	
**29**	RPS6KA6	MAPK	[34]	
**30**	ZAK	MAPK		
**31**	NFATC2	MAPK, Wnt	[35]	
**32**	CXXC4	Wnt		
**33**	FZD3	Wnt	[36,37]	
**34**	SMAD2	Wnt	[19,38]	
**35**	SMAD4	Wnt	[38,39]	
**36**	VANGL2	Wnt	[40]	

Supplementary Table 15. Primers: Complete list of primers used for qRT-PCR experiments

Gene	TaqMan Assay ID
**miR-146a-5p**	#000468
**miR-146b-5p**	#001097
**miR-24-3p**	#000402
**miR-425-3p**	#002302
**ACVR1B**	Hs00244715_m1
**EGFR**	Hs01076090_m1
**FGF2**	Hs00266645_m1
**MAP3K8**	Hs00178297_m1
**MAPK14**	Hs01051152_m1
**MAPK8**	Hs01548508_m1
**MAPT**	Hs00902194_m1
**MRAS**	Hs00171926_m1
**NFATC2**	Hs00905451_m1
**NTRK2 (T1)**	Hs01093110_m1
**PDGFRA**	Hs00998026_m1
**PDGFRB**	Hs01019589_m1
**PPM1A**	Hs01056778_g1
**PRKACA**	Hs00427274_m1
**PRKACB**	Hs01086757_m1
**PRKCA**	Hs00925193_m1
**PRKCB**	Hs00176998_m1
**RAC1**	Hs01902432_s1
**RASGRF1**	Hs00182314_m1
**RPS6KA3**	Hs00177936_m1
**RPS6KA6**	Hs00179523_m1

SUPPLEMENTARY REFERENCES

1. Lavebratt, C., Aberg, E., Sjoholm, L. K. & Forsell, Y. Variations in FKBP5 and BDNF genes are suggestively associated with depression in a Swedish population-based cohort. Journal of affective disorders 125, 249-255, doi:10.1016/j.jad.2010.02.113 (2010).
2. Drago, A. et al. AKAP13, CACNA1, GRIK4 and GRIA1 genetic variations may be associated with haloperidol efficacy during acute treatment. European neuropsychopharmacology : the journal of the European College of Neuropsychopharmacology 23, 887-894, doi:10.1016/j.euroneuro.2012.08.013 (2013).
3. Romano, A. et al. Depressive-like behavior is paired to monoaminergic alteration in a murine model of Alzheimer's disease. Int J Neuropsychopharmacol 18, doi:10.1093/ijnp/pyu020 (2015).
4. Maussion, G. et al. Regulation of a truncated form of tropomyosin-related kinase B (TrkB) by Hsa-miR-185* in frontal cortex of suicide completers. PLoS One 7, e39301, doi:10.1371/journal.pone.0039301 (2012).
5. Wang, J. W., Dranovsky, A. & Hen, R. The when and where of BDNF and the antidepressant response. Biol Psychiatry 63, 640-641, doi:10.1016/j.biopsych.2008.01.008 (2008).
6. Ernst, C., Chen, E. S. & Turecki, G. Histone methylation and decreased expression of TrkB.T1 in orbital frontal cortex of suicide completers. Mol Psychiatry 14, 830-832, doi:10.1038/mp.2009.35 (2009).
7. Kruk, J. S. et al. Fluoxetine-induced transactivation of the platelet-derived growth factor type beta receptor reveals a novel heterologous desensitization process. Molecular and cellular neurosciences 65, 45-51, doi:10.1016/j.mcn.2015.02.013 (2015).
8 .Malki, K. et al. Convergent animal and human evidence suggests a role of PPM1A gene in response to antidepressants. Biol Psychiatry 69, 360-365, doi:10.1016/j.biopsych.2010.08.011 (2011).
9. Xu, F. et al. Differential co-expression and regulation analyses reveal different mechanisms underlying major depressive disorder and subsyndromal symptomatic depression. BMC bioinformatics 16, 112, doi:10.1186/s12859-015-0543-y (2015).
10. Nestler, E. J. Role of the Brain's Reward Circuitry in Depression: Transcriptional Mechanisms. International review of neurobiology 124, 151-170, doi:10.1016/bs.irn.2015.07.003 (2015).
11. Shelton, R. C., Hal Manier, D. & Lewis, D. A. Protein kinases A and C in post-mortem prefrontal cortex from persons with major depression and normal controls. Int J Neuropsychopharmacol 12, 1223-1232, doi:10.1017/S1461145709000285 (2009).
12. Carroll, L. S. et al. Evidence for rare and common genetic risk variants for schizophrenia at protein kinase C, alpha. Mol Psychiatry 15, 1101-1111, doi:10.1038/mp.2009.96 (2010).
13. Costas, J. et al. Association study of 44 candidate genes with depressive and anxiety symptoms in post-partum women. J Psychiatr Res 44, 717-724, doi:10.1016/j.jpsychires.2009.12.012 (2010).
14. Sakaida, M. et al. Electroconvulsive seizure-induced changes in gene expression in the mouse hypothalamic paraventricular nucleus. J Psychopharmacol 27, 1058-1069, doi:10.1177/0269881113497612 (2013).
15. Golden, S. A. et al. Epigenetic regulation of RAC1 induces synaptic remodeling in stress disorders and depression. Nature medicine 19, 337-344, doi:10.1038/nm.3090 (2013).
16. Wei, Y. B., Backlund, L., Wegener, G., Mathe, A. A. & Lavebratt, C. Telomerase dysregulation in the hippocampus of a rat model of depression: normalization by lithium. Int J Neuropsychopharmacol 18, pyv002, doi:10.1093/ijnp/pyv002 (2015).
17. Ploski, J. E., Newton, S. S. & Duman, R. S. Electroconvulsive seizure-induced gene expression profile of the hippocampus dentate gyrus granule cell layer. Journal of neurochemistry 99, 1122-1132, doi:10.1111/j.1471-4159.2006.04156.x (2006).
18. Kim, S. & Webster, M. J. Integrative genome-wide association analysis of cytoarchitectural abnormalities in the prefrontal cortex of psychiatric disorders. Mol Psychiatry 16, 452-461, doi:10.1038/mp.2010.23 (2011).
19. Park, S. H. et al. Melittin inhibits TGF-beta-induced pro-fibrotic gene expression through the suppression of the TGFbetaRII-Smad, ERK1/2 and JNK-mediated signaling pathway. The American journal of Chinese medicine 42, 1139-1152, doi:10.1142/S0192415X14500712 (2014).
20. Ganea, K. et al. Convergent animal and human evidence suggests the activin/inhibin pathway to be involved in antidepressant response. Translational psychiatry 2, e177, doi:10.1038/tp.2012.104 (2012).
21. Bergstrom, A., Jayatissa, M. N., Thykjaer, T. & Wiborg, O. Molecular pathways associated with stress resilience and drug resistance in the chronic mild stress rat model of depression: a gene expression study. Journal of molecular neuroscience : MN 33, 201-215 (2007).
22. Malki, K. et al. Antidepressant-dependent mRNA changes in mouse associated with hippocampal neurogenesis in a mouse model of depression. Pharmacogenetics and genomics 22, 765-776, doi:10.1097/FPC.0b013e328356fa90 (2012).

23. Drago, A., Cocchi, E., Crisafulli, C. & Serretti, A. A molecular pathway analysis of the glutamatergic-monoaminergic interplay serves to investigate the number of depressive records during citalopram treatment. J Neural Transm (Vienna) 122, 465-475, doi:10.1007/s00702-014-1267-2 (2015).
24. Tian, W. et al. A study of the functional significance of epidermal growth factor in major depressive disorder. Psychiatric genetics 22, 161-167, doi:10.1097/YPG.0b013e3283539550 (2012).
25. Yamamori, H. et al. Assessment of a multi-assay biological diagnostic test for mood disorders in a Japanese population. Neuroscience letters 612, 167-171, doi:10.1016/j.neulet.2015.12.019 (2016).
26. Birey, F. et al. Genetic and Stress-Induced Loss of NG2 Glia Triggers Emergence of Depressive-like Behaviors through Reduced Secretion of FGF2. Neuron 88, 941-956, doi:10.1016/j.neuron.2015.10.046 (2015).
27. Borroto-Escuela, D. O., Tarakanov, A. O. & Fuxe, K. FGFR1-5-HT1A Heteroreceptor Complexes: Implications for Understanding and Treating Major Depression. Trends in neurosciences 39, 5-15, doi:10.1016/j.tins.2015.11.003 (2016).
28. Schwarz, R. et al. A preliminary study on methylphenidate-regulated gene expression in lymphoblastoid cells of ADHD patients. The world journal of biological psychiatry : the official journal of the World Federation of Societies of Biological Psychiatry 16, 180-189, doi:10.3109/15622975.2014.948064 (2015).
29. Onwuameze, O. E. et al. MAPK14 and CNR1 gene variant interactions: effects on brain volume deficits in schizophrenia patients with marijuana misuse. Psychological medicine 43, 619-631, doi:10.1017/S0033291712001559 (2013).
30. Elsayed, M. et al. Antidepressant effects of fibroblast growth factor-2 in behavioral and cellular models of depression. Biol Psychiatry 72, 258-265, doi:10.1016/j.biopsych.2012.03.003 (2012).
31. Sathyanesan, M. et al. A molecular characterization of the choroid plexus and stress-induced gene regulation. Translational psychiatry 2, e139, doi:10.1038/tp.2012.64 (2012).
32. Fabbri, C. & Serretti, A. Genetics of long-term treatment outcome in bipolar disorder. Progress in neuro-psychopharmacology & biological psychiatry 65, 17-24, doi:10.1016/j.pnpbp.2015.08.008 (2016).
33. Darcy, M. J., Trouche, S., Jin, S. X. & Feig, L. A. Age-dependent role for Ras-GRF1 in the late stages of adult neurogenesis in the dentate gyrus. Hippocampus 24, 315-325, doi:10.1002/hipo.22225 (2014).
34. Smagin, D. A. et al. Dysfunction in Ribosomal Gene Expression in the Hypothalamus and Hippocampus following Chronic Social Defeat Stress in Male Mice as Revealed by RNA-Seq. Neural plasticity 2016, 3289187, doi:10.1155/2016/3289187 (2016).
35. Inkster, B. et al. Pathway-based approaches to imaging genetics association studies: Wnt signaling, GSK3beta substrates and major depression. NeuroImage 53, 908-917, doi:10.1016/j.neuroimage.2010.02.065 (2010).
36. Hashimoto, R. et al. Association study of the frizzled-3 (FZD3) gene with schizophrenia and mood disorders. J Neural Transm (Vienna) 112, 303-307, doi:10.1007/s00702-004-0264-2 (2005).
37. Keri, S., Szabo, C. & Kelemen, O. Blood biomarkers of depression track clinical changes during cognitive-behavioral therapy. Journal of affective disorders 164, 118-122, doi:10.1016/j.jad.2014.04.030 (2014).
38. Dow, A. L., Russell, D. S. & Duman, R. S. Regulation of activin mRNA and Smad2 phosphorylation by antidepressant treatment in the rat brain: effects in behavioral models. The Journal of neuroscience : the official journal of the Society for Neuroscience 25, 4908-4916, doi:10.1523/JNEUROSCI.5155-04.2005 (2005).
39. Suda, S., Segi-Nishida, E., Newton, S. S. & Duman, R. S. A postpartum model in rat: behavioral and gene expression changes induced by ovarian steroid deprivation. Biol Psychiatry 64, 311-319, doi:10.1016/j.biopsych.2008.03.029 (2008).
40. Okerlund, N. D. & Cheyette, B. N. Synaptic Wnt signaling-a contributor to major psychiatric disorders? Journal of neurodevelopmental disorders 3, 162-174, doi:10.1007/s11689-011-9083-6 (2011).
